# Orthography-phonology consistency in English: Theory- and data-driven measures and their impact on auditory vs. visual word recognition

**DOI:** 10.3758/s13428-023-02094-5

**Published:** 2023-08-08

**Authors:** Alfred Lim, Beth O’Brien, Luca Onnis

**Affiliations:** 1 School of Psychology, University of Nottingham Malaysia, Semenyih, Selangor, Malaysia; 2 Centre for Research in Child Development (CRCD), National Institute of Education, Singapore, Singapore; 3 Centre for Research and Development on Learning (CRADLE), Nanyang Technological University, Singapore, Singapore; 4 Centre for Multilingualism in Society across the Lifespan, University of Oslo, Semenyih, Selangor Malaysia; 5 Department of Linguistics and Scandinavian Studies, University of Oslo, Oslo, Norway

**Keywords:** Spelling-sound consistency, Sound-spelling consistency, Word naming, Lexical decision, Word recognition, Computational modelling

## Abstract

Research on orthographic consistency in English words has selectively identified different sub-syllabic units in isolation (grapheme, onset, vowel, coda, rime), yet there is no comprehensive assessment of how these measures affect word identification when taken together. To study which aspects of consistency are more psychologically relevant, we investigated their independent and composite effects on human reading behavior using large-scale databases. Study 1 found effects on adults’ naming responses of both feedforward consistency (orthography to phonology) and feedback consistency (phonology to orthography). Study 2 found feedback but no feedforward consistency effects on visual and auditory lexical decision tasks, with the best predictor being a composite measure of consistency across grapheme, rime, OVC, and word-initial letter-phoneme. In Study 3, we explicitly modeled the reading process with forward and backward flow in a bidirectionally connected neural network. The model captured latent dimensions of quasi-regular mapping that explain additional variance in human reading and spelling behavior, compared to the established measures. Together, the results suggest interactive activation between phonological and orthographic word representations. They also validate the role of computational analyses of language to better understand how print maps to sound, and what properties of natural language affect reading complexity.

## Introduction

The ability to recognize written representations of words is considered foundational for fluent reading acquisition and comprehension. As a pivotal process in literacy word reading has been the focus of an extensive body of psycholinguistic research. For skilled adult readers, this research points to the well-specified representations of words’ phonology, orthography, and meaning within the mental lexicon (Perfetti, 2007). While there is agreement that in order to acquire and master such decoding abilities readers must learn to map between orthography (print) and phonology (speech) (Verhoeven & Perfetti, 2017), the specific properties of writing systems that are most cognitively relevant to the reading brain have not been entirely spelled out.

Skilled readers of alphabetic languages are able to ‘cipher’ or decode known and unfamiliar words using acquired orthographic-phonological mappings (Ehri & Wilce, 1987), otherwise referred to as grapheme–phoneme correspondences (GPCs), where ‘graphemes’ refer to single letters or letter clusters that correspond to a single ‘phoneme’ or speech sound. Readers are also adept spellers, and so they have also acquired phoneme-grapheme correspondences (PGCs). To establish these mapping systems (GPCs and PGCs), beginning readers take into account the statistical regularities implicit in the written and spoken language, and the regularities of the correspondences between them.

Regularities can occur in multiple guises, for example in the way that phonemes are combined within spoken words—phonotactic regularities. For instance, the phoneme /ŋ/ appears only at the end of words in English, but at the beginning of words in Swahili. Such phonological regularities often appear reflected in written words—as orthotactic and graphotactic regularities. For example, the letter sequence *NG* also appears at the end of English words but not at the beginning, and noticing this regularity can help the learner map onto the phoneme /ŋ/.

However, orthographic systems are often compromise solutions between print and sound, as they are the historical product of layered adaptations, idiosyncratic habits handed down and becoming conventionalized over centuries, and consequences of language contact. For example, the Roman alphabet script originally containing 23 letter symbols was progressively adopted by several languages in Europe and beyond, with fairly different phonemic systems and inventories. When the Anglo-Saxons, linguistic ancestors of English speakers, adopted the Roman alphabet to correspond with the sounds of their own language, they had to confront the fact that the alphabet contained only five graphemes to indicate vowels, while today’s English varieties contain at least 21 phonemic vowels. Because of multiple historical facts such as these, for any given natural language the print-sound mappings—and thus the underlying statistics upon which learning occurs—can be more or less regular. For instance, the grapheme *NG* also occurs in the middle of English words to map to a different set of phonemes /ndʒ/, as in the word *ENGINEER*. Or the grapheme *CH* can map onto three different phonemes: /k/ as in CHAOS, /ʃ/ as in MACHINE, and /tʃ/ as in CHINA. More consistent orthographies, like Finnish or Italian, exhibit fewer and more regular GPC and PGC patterns than English, and thus an overall more economical mapping between print and sound. For instance, the grapheme CH maps onto a single phoneme /k/ in Italian. Less consistent orthographies contain more quasi-regularities, where one grapheme can match to more than one phoneme, or phonemes can have inconsistent spellings.

One direct consequence of varying degrees of consistency is that reading is acquired at a comparatively slower rate for readers of more inconsistent graphophonemic systems (Ellis & Yuan, 2004; Georgiou, Parrila, & Papadopoulos, 2008; Florit & Cain, 2011; Frith, Wimmer, & Landerl, 1998). Moreover, within any alphabetic language, more consistent words are read faster and more accurately (Jared, 2002), and this principle also applies to words within more consistent orthographies (Ventura, Morais, Pattamadilok, & Kolinsky, 2004). Thus, besides identifying scripts with more opacity and inconsistencies, it is important to better understand and identify the degree of consistency/inconsistency of words within a language’s script, and how it affects word recognition. In the present study, we examined to what extent the accuracy and latency of word recognition from a large collection of adult participant responses is affected by various measures of print-speech consistency. While our method was applied to English and native speakers of English, we documented and share all procedures and computational pipelines, so that they could be readily applied to other alphabetic systems in future studies.

### The current study

The first goal in this paper was to review several dimensions of word consistency proposed in the literature, and subsequently assess which best accounts empirically for the ease or difficulty of word reading by experienced adult readers. We quantified sublexical features that make English words more or less regular in orthography-to-phonology and phonology-to-orthography mappings.

Because these measures have been mostly studied individually, we asked whether a word-level combined measure captures more systematic psycholinguistic behavior in word identification. Mapping print-sound regularities can occur at different levels of granularity, both from spelling-to-sound (e.g., Hino & Lupker, 1996; Stanovich & Bauer, 1978; Waters & Seidenberg, 1985), and in the opposite direction of sound-to-spelling (e.g., Balota, Cortese, Sergent-marshall, Spieler, & Yap, 2004; Chee, Chow, Yap, & Goh, 2020; Ziegler et al., 1997b). We perused the literature for the various measures proposed and calculated them for thousands of words in a large and representative corpus of English.

The second goal of this paper was thus to ask whether the contribution of orthography-to-phonology and phonology-to-orthography mappings differ depending on the lexical task at hand, i.e., when it is based on visual processing (such as naming or recognizing a written word), and when it is based on auditory processing (such as recognizing a spoken word). To do so, we directly compared the degree of fit of orthography-to-phonology and phonology-to-orthography consistency measures in predicting behavioral visual response data from the English Lexicon Project (ELP, Balota et al., 2007; see Study 1) against data from a new large auditory and production dataset (the Massive Auditory Lexical Decision, MALD, Tucker et al., (2019); see Study 2).

In particular, in Study 1, we analyzed behavioral data from the ELP, which contains behavioral naming response times and accuracy to a naming task of North American English. Based on previous findings, we hypothesized that consistency defined at different granularities shows only moderate overlap, and that a combined measure of consistency across granularity and mapping direction should explain more variance in visual word-recognition performance than individual components (Siegelman, Kearns, & Rueckl, 2020). We found that a composite measure of feedback consistency best accounted for word naming latencies.

In Study 2, we applied the same corpus-derived measures of word consistency to predicting word-recognition performance on a different word task—lexical decision—in both the visual and auditory modalities. Following prior studies, we hypothesized that feedforward consistency should facilitate visual lexical decision performance (Jared, 2002), while feedback consistency should facilitate auditory lexical decision (Grainger & Ziegler, 2011). However, we found only feedback consistency measures best predicting visual lexical decision times.

By the end of Study 2, two considerations became apparent, and we decided to tackle them in Study 3. One consideration is that several dimensions of statistical quasi-regularities between orthography and phonology embedded in the (English) lexicon may be subtle enough to be unaccounted for by the measures used in Study 1 and 2, as in general they may be difficult to identify entirely in researcher-driven analysis. Such undetected patterns of sub-regularity may account for unexplained variance in lexical processing. We thus asked whether a data-driven, machine learning approach implemented in neural networks could contribute to improved overall measures of GPC and PGC consistencies for English words. Modeling reading processes with neural networks has an established tradition since the seminal work of Seidenberg and McClelland (1989), and dovetails with a growing body of empirical evidence that characterizes learning to decode printed words as a form of statistical learning. Because the neural networks we implemented incorporate algorithms of statistical learning and were not taught orthography-phonology mappings explicitly, they represent valid candidate models of what could be learned implicitly from printed words, and how a data-driven approach resolves the mapping problem. In Study 3, we asked whether this data-driven approach to word consistencies provides a better predictor of lexical decision performance than the corpus-derived measures of consistency.

A second consideration for modeling consistency using neural networks is of theoretical relevance and emerged from Study 1 and 2. We found that processes of word identification may rely on resonant bidirectional flows of information relating print to sound and sound to print, perhaps more than has been acknowledged in the literature. This was evident in sound-to-print effects in both the naming word task and the lexical decision tasks, both visual and auditory.

Neural networks lend themselves naturally to modeling interactive effects directly, when forward and backward information flow is implemented explicitly in architectures that are bidirectionally connected. Therefore, we set to train bidirectional neural networks on orthographic-to-phonological mappings (thus simulating reading aloud visually presented words) as well as on phonological-to-orthographic mappings (thus simulating spelling spoken words). The ease and accuracy of the models in solving the mapping problem after training provides a natural alternative metric of word consistency: that is, the closeness to the target phonological word when the network is prompted with an orthographic word as input, and vice versa, the closeness to the target orthographic word when the network is prompted with a phonological input word. In a final set of regression analyses aimed at predicting the human behavioral performance in naming and lexical decision tasks, we compared the fit of our best research-driven consistency predictors (from Study 1 and 2) with the data-driven, neural network consistency predictors obtained in Study 3. To the extent that these networks are bidirectionally connected, they should maximally extract latent quasi-regularities while learning to associate print to sound and vice versa. As a consequence, their performance on individual words could be used to predict human lexical decision performance to a greater sensitivity than the corpus-derived measures of consistency obtained in Study 1 and 2. If neural networks indeed provide a better fit to the human data, we argue that the consistency metrics extracted from their training should be considered as a valid holistic measure of individual words’ consistency in psycholinguistic research. The practical value of this approach should not go unnoticed, as training neural networks has become reasonably fast with modern computers. Therefore, obtaining word-level consistency measures across different languages would be conveniently less resource-intensive, at least compared to the manual hand-picking procedure necessary to identify and extract hundreds of language-specific GPC and PGC mappings (as in Study 1 and Study 2).

Finally, a third goal of this paper was to make available to the scientific community empirical measures of word consistency that can be adopted as a benchmark for future research studies, both experimental and computational, as well as for educational purposes. We share our data publicly in the hope that it can be incorporated in current and next generation psycholinguistic datasets. From an educator’s standpoint, knowing which sets of words may be problematic to learn would allow one to order instruction in line with such challenges, and knowing which patterns of consistent sub-regularities can be capitalized on would likewise help reading instruction. Thus, educational researchers and educators may find useful the ranking of English words in terms of their statistical consistency using the single composite metrics we obtained, when selecting words for experimental tests or to introduce them at different stages of the school curriculum. The resource we offer can thus have both scientific and educational value.

In sum, in the studies that follow we extracted from language corpora consistency measures defined across (a) different sublexical units, and (b) different print-sound direction (feedforward, feedback) and the goal was to find what measure best predicts human performance in (c) three word-recognition tasks. The three studies combined contribute to characterizing the statistical structure of English words in relation to mapping print to sound and sound to print.

## Corpus-derived estimates of reading consistency

In this section, we review dimensions of quasi-regularity that have been advanced in the literature, and empirically calculate corpus-derived measures of such regularities for a sizeable portion of English words. A common way of framing the concept of regularity is to consider alphabetic reading as involving identifying words that follow typical spelling-sound patterns, or rules, but also words that do not adhere to these rules. Therefore, to balance the two demands of alphabetic reading, the reader must generalize the rules to ’consistent’ words, and also learn the exceptions of ‘inconsistent’ words. This has been extensively examined in the psycholinguistic literature (Fodor & Pylyshyn, 1988; Glushko, 1979; Taylor, Plunkett, & Nation, 2011). In one area of research, the distinction is made between categories of regular words that follow spelling rules (e.g., MIST), and irregular words that do not (Castles & Coltheart, 1993, YACHT;). One theoretical approach proposes that each category is handled by two separate cognitive processes—applying GPC rules to decode regular words, or using a mental lexical lookup table to identify irregular words (Coltheart, Rastle, Perry, Langdon, & Ziegler, 2001).

Other theoretical work considers consistency as a continuum dimension (Jared, 1997), whereby words can more or less follow similar pronunciations from similar spellings. For example, in English, the letter N often denotes the phoneme /n/, but letter combinations containing non-pronounced letters such as KN and GN also denote this phoneme as in KNOW, KNEE, GNAT, SIGN, and so on. From the perspective of an implicit learner, such mappings are informative sub-regularities rather than random “exceptions” (Arciuli, 2018). Indeed, degrees of word consistency affect word naming and lexical decision times for adult readers, with faster responses for consistent words (Andrews, 1982; Jared, 1997; 2002). Children also show better accuracy for reading and spelling of consistent words (Alegria & Mousty, 1996; L’et’e, Peereman, & Fayol, 2008; Weekes, Castles, & Davies, 2006). Thus, consistency as a continuum is an important factor within the language, just as it is between shallow and deep alphabetic languages (the orthographic depth hypothesis, Katz and Feldman (1983)).

In the literature, consistency has been configured in different ways (Borleffs, Maassen, Lyytinen, & Zwarts, 2017). Here, we aim to review them separately and then consider them jointly to establish a combined measure of consistency for English words. In some cases, consistency of words has been computed at the grapheme level (Berndt, Reggia, & Mitchum, 1987), whereby the various pronunciations of a grapheme are tabulated across a corpus of words. For example, graphemes often have more than one possible pronunciation (e.g., E → /ɛ/, E → /i/, E → /ə/), and consistency is defined by the variability of the pronunciations assigned to a particular graphemic unit (a single letter, A, or cluster of letters, AY). A word’s consistency can then be taken as an aggregate of a word’s grapheme consistency levels. Others have defined consistency at the subword level for rime spelling patterns (Jared, 1997), which is the vowel nucleus plus any ending consonants. In this case, there are “friends” which are words with shared rime spellings and their pronunciations (HINT, MINT, TINT), and “enemies” which are words that have similar rime spelling but different pronunciations (PINT). A word’s consistency is thus calculated as a ratio between friends and enemies (Jared, 1997). Still, another proposed way to compute consistency involves all subword components, namely onset (initial graphemes coming before the vowel), vowel (nucleus), and the coda (ending graphemes coming after the vowel; Kessler & Treiman, 2001).

**Fig. 1:**
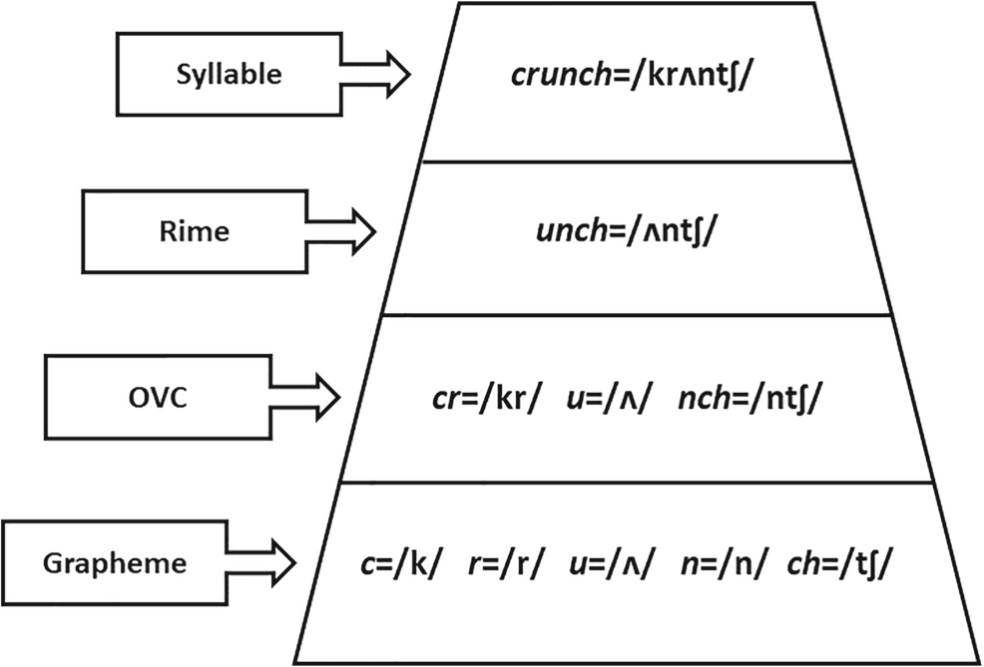
An illustration of the hierarchy of psycholinguistic units in printed words, and how they map to phonological units. Adapted from Ziegler and Goswami (2005)

Thus, different psycholinguistic units have been postulated as the basis for determining word consistency: from grapheme units, to subsyllabic onset-vowel-coda units, to rime patterns (as shown in Fig. 1). For the beginning reader, the process of decoding words from these print units to mapped speech units requires first a segmenting process, which is non-trivial. Delineating subword patterns is complicated by the fact that units corresponding to a single phoneme also differ in granularity, or the number of letters that are contained in the graphemic unit. Subword patterns become unitized for experienced readers, as demonstrated when adults are slower to identify individual letters within a multi-letter grapheme (Rey, Ziegler, & Jacobs, 2000). So another essential part of learning to read involves this process of unitization. On the other hand, the mapping process involves pronunciation variability which may be affected by word context, such as non-sequential letter patterns, like the silent vowel E which can affect the pronunciation of the previous vowel (e.g., PLANE → /pleɪn/, instead of /plæni/). Both granularity and consistency, then, are important aspects of language structure that impact reading acquisition and performance.

As defined above, consistency may depend on the level of the units for which it is evaluated. For example, rime patterns are held to play an important role in the pronunciation of printed words (Treiman, Mullennix, Bijeljac-babic, & Richmond-Welty, 1995). Consider the word PINT (/paɪnt/). At the rime level, it is an inconsistent word because it is pronounced differently than other words sharing its rime spelling pattern, like MINT (/mɪnt/) and TINT (/tɪnt/), and these two mappings have different probabilities (INT → /aɪnt/, *p* = 0.04, versus INT → /ɪnt/, *p* = 0.91). Yet, at the grapheme level PINT (/paɪnt/) has an overall predictability across its graphemes of *p* = 0.87 (P → /p/, *p* = 1.00; I → /aɪ/, *p* = 0.49; N → /n/, *p* = 1.00; T → /t/, *p* = 1.00), calculated based on the average of the ratio of each GPC probability and the most probable correspondence for that combination (Berndt et al., 1987).

Even in cases where the rime pattern is consistently pronounced across words (such as AND → /ʌnd/, *p* = 0.92), its vowel is often inconsistent across words (A → /ʌ/, *p* < 0.01). Siegelman et al., (2020) address this important issue for operationalizing consistency, and suggest alternative methods focused on uncertainty using information theory, as described below. Here, we compare different methods previously used for deriving consistency.

We first apply these various definitions of consistency across a word corpus and examine their interrelations, along with a new integrated measure of consistency. We then examine how well the different measures of consistency predict recorded human response times for visual word processing (from the ELP database, Balota et al., 2007) and then in Study 2 additionally for auditory processing (from the MALD database, version 1.1, Tucker et al., (2019)). The ELP contains behavioral data from 1260 participants across six different universities who responded to 40,000 words in a visual naming task and a visual lexical decision task, while the MALD database comprises response data for 26,793 words and 9592 pseudowords in a auditory lexical decision task from 231 unique monolingual English listeners.

### Method

#### Corpus

For the present study, we selected only monosyllabic words from the Massive Auditory Lexical Decision (MALD) database (Tucker et al., 2019) (*N* = 4,347) to derive and compare their consistency. We used the subtitle-based SUBTLEX-US (Brysbaert, New, & Keuleers, 2012) frequency measure to compute frequency-weighted consistency measures. Tucker et al., (2019) previously found that the SUBTLEX-US frequency count best explains frequency effects on response times when compared to the Corpus of Contemporary American English (COCA ; Davies, 2009) and Google Books n-gram corpus.

The MALD database is a freely available auditory data set for psycholinguistic research, providing time-aligned stimulus recordings for 26,793 words and 9592 pseudowords, and response data for 227,179 auditory lexical decisions from 231 unique monolingual English listeners.

### Consistency at different granularities

To capture multiple levels of consistency for each word more holistically, we computed four sub-level consistency measures proposed by Berndt et al., (1987), Jared (1997), Kessler and Treiman (2001), Borgwaldt, Hellwig, and De Groot (2005), and corresponding to the grapheme, rime, onset-vowel-coda (OVC), and the onset level, respectively (see Fig. 1).

#### Grapheme consistency

The first measure captures word consistency at the grapheme level (referred to as *grapheme consistency* from here onwards ;Berndt et al., 1987), which requires the probabilities of grapheme–phoneme associations to first be computed as they occur in the corpus (e.g., the probability of the grapheme EW being pronounced as /o/ is, *p*(/o/|*EW*) = 0.06). Using these probabilities, the overall consistency of a word’s pronunciation is defined as the average of the ratio of each probability (e.g., *p*(/o/|*EW*) = 0.06) and the most probable correspondence for that grapheme (e.g., *p*_*max*_(*EW*) = 0.94). For example, the overall grapheme consistency predictability for the word SEW is calculated by taking the ratio average of the graphemes S(*p*(/s/|*S*) = 0.63 / *p*_*max*_(*S*) = 0.63) and EW (*p*(/oʊ/|*EW*) = 0.63 / *p*_*max*_(*EW*) = 0.94), resulting in the value 0.83.

#### Rime consistency

The second measure is at the orthographic rime level (referred to as the *rime consistency* from here onwards; Jared (1997)). It is calculated as the proportion of *friends* and *enemies* amongst words that are similar orthographically in that they share vowel and coda spellings (e.g., the neighborhood: PINT, MINT, TINT). For example, for a word ending in INT, the rime-consistency was defined as the number of *friends* relative to the total number of *friends* plus *enemies*—where a *friend* is a word with the same orthographic rime unit and the same pronunciation of that unit, and an *enemy* is a word with the same orthographic rime unit and a different pronunciation.

#### OVC consistency

The third consistency measure considers the grapheme-to-phoneme consistencies of onset, vowel, and coda of words (referred to as *OVC consistency* from here onwards; Kessler and Treiman (2001)). Kessler and Treiman (2001) proposed a new measure termed *conditional consistencies* that is calculated on one part of the word when we hold constant some other part of the word. For example, one could compute the reading consistency of the vowel letter I when the coda is NT. A total of nine probability values (three unconditional and six conditional probabilities) were computed for each word by taking into account the letter strings of each of the three parts (onset, vowel, coda) and the combinations of any of the two parts (e.g., onset-vowel, onset-coda) of the syllable.

#### Onset consistency

The last measure focuses on the onsets of words and computed the consistency for word-initial letter-to-phoneme correspondences. Onset-consistency has been found to influence reaction times in reading tasks (Glushko, 1979; Treiman et al., 1995) and plays an important role in lexical access tasks (Marslen-Wilson & Welsh, 1978; Marslen-Wilson & Zwitserlood, 1989). Here, we considered the different pronunciations of first letters as in Borgwaldt et al., (2005) and computed the extent to which words with the same first letter also have the same first phoneme. For example, English words that begin with the letter W may have a different first phoneme: /w/ as in WING, *p*(/w/|*W*) = 0.94; /r/ as in WRAP, *p*(/r/|*W*) = 0.05; and /h/ as in WHOM, *p*(/r/|*W*) = 0.06.

### From probabilities to information-theoretic measures

The conditional probabilities described above were later converted to surprisal, entropy, and information gain (*IG*) bits—indices borrowed from information theory (see also Siegelman et al., 2020).

*Surprisal* captures the unpredictability of a given grapheme-to-phoneme correspondence and, unlike probability, makes fine distinctions between low and very low probabilities via a non-linear logarithmic transformation:

1 $$  S_{i} = -log_{2} p(i) $$

where *p*(*i*) is the probability of an event *i* (e.g., *p*(/o/|*EW*)). Contrary to probability, higher surprisal values represent more surprising pronunciations, and it has been found to predict behavioral indices of language processing difficulty better than probability (e.g., Smith and Levy, 2013).

*Entropy* captures the unpredictability in the distribution of possible pronunciations of an event (e.g., how unpredictable a grapheme is given all its possible pronunciations) and is computed summing the surprisal of each event (*S*_*i*_) multiplied by the probability of the event’s occurrence [*p*(*i*)]:

2 $$  E = -\sum\limits_{i}p(i) * log_{2} p(i) $$

Entropy was first introduced by Shannon’s information theory (Shannon, 1948), and earlier psycholinguistic studies have used entropy to investigate processing difficulty in human sentence comprehension (e.g., Levy, 2008).

Lastly, *IG* was computed for each word by finding the difference between entropy and surprisal (*E* − *S*), which quantifies the predictability of a grapheme-to-phoneme correspondence given the unpredictability of the grapheme. All analyses were performed on IG bits from here onwards.

### Feedforward and feedback consistency

Typically, the mapping from pronunciation to spelling is less consistent than the mapping from spelling to pronunciation, and this may be one reason why spelling tasks are more difficult than reading tasks. Studies of word identification reveal that reading times are longer for words containing a sequence of phonemes that can be spelled in multiple ways. For example, it has been reported that adults are slowed when reading a word like HURL because other words that HURL rhymes with, such as GIRL and PEARL, have different spellings of the same rhyme (e.g., Lacruz & Folk, 2004; Stone, Vanhoy, & Van Orden, 1997; Ziegler, Montant, & Jacobs, 1997a; Perry, 2003). This form of inconsistency in the sound-to-letter direction, as opposed to letter-to-sound direction, is often referred to as the feedback consistency effect, which was first demonstrated by Stone, Vanhoy, and Van Orden (1997).

The theoretical implication of these findings suggests that reading words does not depend solely on converting an orthographic form into a phonological representation, but the process also involves a feedback mechanism from phonology to orthography to verify that the phonological representation can be spelled in that orthographic form. It is therefore believed that spelling and reading are intimately related and may influence each other during word processing. That is, both reading and spelling tasks can be affected by the combination of feedforward and feedback consistency.

The procedure used to compute the four-level consistency measures (i.e., grapheme, rime, OVC, onset) in the GPC direction was repeated using PGCs (for spelling). Separate GPC and PGC conditional probabilities were calculated using the same sound-letter components in the corpus. Taking the word PINT for example, its GPC conditional probability (INT → /aɪnt/, *p* = .04) and PGC conditional probability (/aɪnt/ → INT, *p* = 1.0) derived using the *rime consistency* method were based on the same rime and phonemes, differing only in the direction of correspondence.

### Word-level consistency

Once sub-level consistency measures have been computed, we further derived three word-level measures using *composite score*, *principal component*, and *least consistent unit* by taking all four sub-level measures into account, with a higher score representing higher overall word consistency.

#### Composite score

As mapping print-sound regularities can occur at different levels of granularity, consistency has, too, been defined differently in the literature, which often resulted in inconsistent findings. Therefore, it is necessary to combine the various unit-level measures to obtain a combined index of word consistency. One method is to use a simple mean (unweighted) composite score that averages across the four unit-level measures.

#### Principal component analysis

Second, we made use of principal component analysis (PCA) for dimensionality reduction, and extracted the first principal component (PC1) for a maximal amount of total variance in the variables. Our results showed that the PC1 of feedforward consistency (FF_PC1) has an eigenvalue of 16, where 73% of the variance was extracted, and the PC1 of feedback consistency (FB_PC1) accounted for 84% of the variance (eigenvalue of 76). Therefore, PC1s were sufficient to account for most variance in the data.

#### Least consistent unit

The previous two composite and PC1 measures are susceptible to extreme values. This is especially profound when a unit (e.g., rime) of a word is highly consistent or inconsistent, while its consistency measured at other units are less extreme. As such, it is important to determine if an observed consistency effect is simply due to the word-level measure being skewed by its most or least consistent unit. To verify this possibility, we extracted the lowest value among all unit-level measures of each word as a word-level consistency measure of its own.

### Corpus analyses

**Fig. 2:**
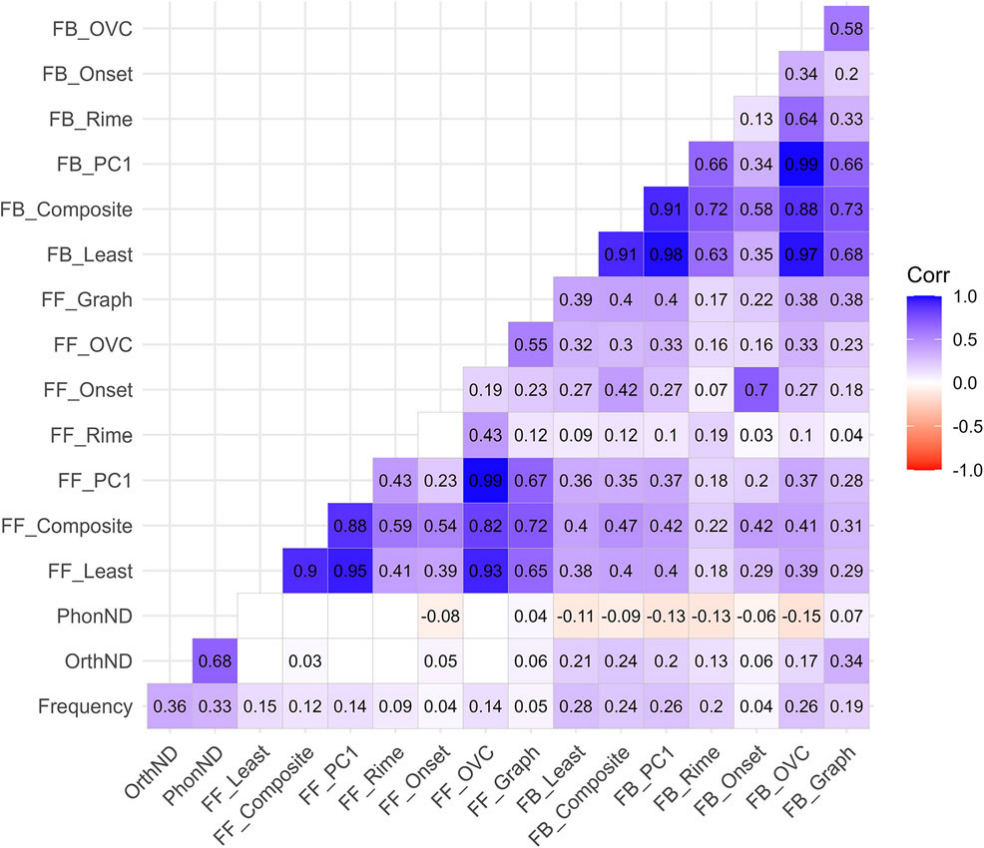
Correlation matrix among the feedforward and feedback consistency measures computed at the unit and word levels. The *darker blue color* denotes a stronger positive correlation, and the *darker red color* denotes a stronger negative correlation. *Numbers* indicate correlation coefficients, and *empty cells* indicate no significant correlation was found (*p* > .05)

This section contains descriptive statistics of the MALD corpus (Tucker et al., 2019). To ascertain that these measures may in fact capture different aspects of consistency, we plotted a correlation matrix of each measure against the others (see Fig. 2) with a description of the labels provided in Table 1 and pre-scaling descriptive statistics presented in Table 2.

**Table 1:** Description of the variables used in the present study

Label	Description
FF_Rime	Feedforward rime consistency (IG)
FF_Onset	Feedforward onset consistency (IG)
FF_OVC	Feedforward OVC consistency (IG)
FF_Graph	Feedforward grapheme consistency (IG)
FF_Least	Lowest score among all four feedforward consistency measures (IG)
FF_Composite	Composite score of feedforward consistency measures (IG)
FF_PC1	First principal component (PC1) of feedforward consistency measures
FB_Rime	Feedback rime consistency (IG)
FB_Onset	Feedback onset consistency (IG)
FB_OVC	Feedback OVC consistency (IG)
FB_Graph	Feedback grapheme consistency (IG)
FB_Least	Lowest score among all four feedback consistency measures (IG)
FB_Composite	Composite score of feedback consistency measures (IG)
FB_PC1	First principal component (PC1) of feedback consistency measures
OrthND	The number of orthographic neighbors (one glyph edit away)
PhonND	The number of phonological neighbors (one phone edit away)
Frequency	Word frequency (SUBTLEX-US corpus; Brysbaert, New, & Keuleers, 2012)

**Table 2:** Descriptive statistics before scaling

	Mean	Median	SD	Range	Skewness
FF_Rime	− 0.27	0.00	1.24	15.37	− 5.54
FF_Onset	− 0.23	0.00	1.37	19.57	− 5.68
FF_OVC	− 1.58	0.00	3.72	39.71	− 2.84
FF_Graph	− 0.35	0.24	2.09	25.65	− 3.92
FF_Least	− 2.24	− 0.42	3.70	37.97	− 2.74
FF_Composite	− 0.00	0.24	0.66	6.31	− 3.28
FF_PC1	− 0.00	1.56	3.99	41.60	− 2.96
FB_Rime	− 1.05	0.00	2.48	19.17	− 2.51
FB_Onset	− 0.10	0.02	1.13	17.87	− 7.30
FB_OVC	− 4.95	− 2.41	8.20	108.60	− 3.08
FB_Graph	− 2.08	− 0.75	3.94	31.00	− 1.72
FB_Least	− 5.90	− 3.60	7.87	105.04	− 3.23
FB_Composite	0.00	0.23	0.73	8.91	− 2.89
FB_PC1	0.00	2.64	8.71	105.34	− 2.82
OrthND	17.37	14.00	13.11	77.00	1.16
PhonND	51.32	40.00	40.55	240.00	1.19
Frequency	5.69	5.50	2.21	13.88	0.58

Results show that while all of the consistency measures were significantly related, there was a wide range of the correlation coefficients. For example, forward OVC- and grapheme-level measures were moderately correlated [*r*(4345) = 0.554, *p* < .001], whereas rime level showed a weaker correlation with grapheme-level consistency [*r*(4345) = 0.119, *p* < .001], suggesting that consistency measured at different sub-levels are related but not identical entirely. Expectedly, many of the feedforward consistency measures were only weakly to moderately related to the feedback consistency measures as they were measured at a different direction [at the rime level, *r*(4345) = 0.189, *p* < .001; OVC level, *r*(4345) = 0.330, *p* < .001; and grapheme level, *r*(4345) = 0.384, *p* < .001; whereas only the onset level showed high correspondence between feedforward and feedback consistency, *r*(4345) = 0.699, *p* < .001;]

Our results indicate that the different approaches to quantifying consistency are not closely aligned. With regard to the derived composite scores, all feedforward consistencies were positively correlated with the feedforward composite score, but to different degrees for the different levels of consistency. Such correlations were greatest when measured at the OVC level [*r*(4345) = 0.817, *p* < .001], followed by grapheme level [*r*(4345) = 0.715, *p* < .001], rime level [*r*(4345) = 0.585, *p* < .001], and onset level [*r*(4345) = 0.536, *p* < .001]. Interestingly, for the feedback composite score, the same ordering was observed with the strongest correlation found at the OVC level [*r*(4345) = 0.880, *p* < .001], followed by grapheme level [*r*(4345) = 0.726, *p* < .001], rime level [*r*(4345) = 0.722, *p* < .001], and onset level [*r*(4345) = 0.576, *p* < .001].

## Study 1: Consistency effects on word naming

In order to establish the extent to which different measures of consistency were differentially predictive of actual human reading behavior, we turned to a dataset of human word naming, the English Lexicon Project (ELP, available at http://elexicon.wustl.edu; see Balota et al., (2007)). From the ELP, we derived 119,214 unique naming reading times (RTs) by 457 different subjects, for the subset of 4207 words shared by the ELP and MALD datasets.

### Procedure

Trial-level RT data were obtained from the ELP database, and trials with an incorrect response were first excluded. Trials with RTs that deviated three times less than the median absolute deviation (MAD) were quantified as “too fast” responses. Likewise, slow outliers were defined as those with RTs three times greater than the MAD. After excluding incorrect trials ($\sim 3.63\%$ of all trials), “too fast” responses ($\sim 0.76\%$), and slow outliers ($\sim 5.30\%$), statistical analyses were performed on the remaining $\sim 90.30\%$ of trials.

Item-level regression analyses (LM) were conducted on the mean RTs for 4207 words for the visual naming task that were obtained from the ELP. The dependent variables consisted of *z*-scored RTs, averaged across participants for each word. Each participant’s raw response times were first standardized using a *z*-score transformation, and the mean *z*-score for all participants presented with a particular word was then computed for that word (Balota et al., 2007). For the analyses of the ELP database, word frequency values were logarithmic transformed to correct for skewness before analysis, similar to that in Balota, Cortese, Sergent-marshall, Spieler, and Yap (2004).

In addition to the lexical variables (e.g., OrthND, PhonND) introduced in our corpus analyses, two binary variables were added to code the initial phoneme of each word. These variables were based on features found to affect response times in Balota et al., (2004), but we coded them into two binary variables to reduce our number of predictors in the regression models. The variable *Onset_Coding* denotes the initial phoneme’s presence or absence (1 = presence, 0 = absence) of any of the following phonological features: nasal, fricative, stop, affricative, and liquid, to control for the variance associated with voice key biases in speeded pronunciation (Balota et al., 2004).

Across age-group and tasks (i.e., naming and lexical decision), Balota et al., (2004) showed that the effects of the 12 phonemic features of onset on RTs were consistent with the exception of voicelessness. Specifically, voicelessness was found to facilitate RTs in naming tasks, but slow RTs in lexical decision tasks. To avoid introducing noise to the Onset_Coding binary variable, we coded voicelessness as a separate binary variable (*Voice*) that denotes if the initial phoneme is voiced or unvoiced (1 = voiced, 0 = unvoiced).

### Analytic approach

First, to compare all the combined and individual measures of consistency, we constructed 14 different predictive models with word naming RTs as the dependent variable, and one of the 14 measures of consistency included as independent predictors in each model. All LM models included seven lexical variables (i.e., Frequency, Num_Phones, OnsetCoding, OrthND, PhonND, Voice, and Word_Length) and one of the derived consistency measures (i.e., feedback and feedforward consistency measures at the rime, onset, OVC, grapheme, and combined levels) as predictors. A baseline model that included only the lexical variables was also added. All predictor variables were standardized (mean = 0, SD = 1) prior to modelling.

Second, based on the model comparison results, we subsequently conducted a two-step hierarchical regression approach to determine if the best word-level measures accounted for additional variance in the word naming RTs over conventional lexical variables. Prior to running the model, multicollinearity was examined using the *Variance Inflation Factor* (VIF) statistics, with lower VIF values indicating low correlations among variables. In Step 1 of the regression analysis, word frequency, number of phonemes, onset coding, number of orthographic neighbors, number of phonological neighbors, onset voicelessness, and word length (Frequency, Num_Phones, OnsetCoding, OrthND, PhonND, Voice, and Word_Length) were entered into the LM model. Depending on the model comparison results, either word-level composites (FB_Composite and FF_Composite), PC1s (FB_PC1 and FF_PC1), or least consistent unit (FB_Least and FF_Least) were entered into the LM model in Step 2, in addition to the previously entered variables.

Third, dominance analyses (DA) were utilized to directly compare the importance and unique contribution of the individual sub-level consistency measures as predictors in the same model, while eliminating the issue of multicollinearity. DA relies on computing *R*^2^ estimates for all possible subset models. Since our models contained a total of eight sub-level consistency measures (i.e., four each from the feedforward and feedback directions), we needed 255 different subset models for all levels of combinations: 8 single predictor models, 28 two-predictor models, 56 three-predictor models, 70 four-predictor models, 56 five-predictor models, 28 six-predictor models, eight seven-predictor models, and one eight-predictor models. A general dominance estimate (Azen and Budescu, 2003) is achieved if a predictor’s unique contribution is greater across the average of all subset models as compared with the competitor predictor.

All statistical analyses were computed with R version 4.0.3 (R Core Team, 2020). The function *lm* in R was used to fit the models using ordinary least squares. Simultaneous information-theoretic model comparison was done using the *model.sel* function in the *MuMin* package (Barton & Barton, 2015), which provides estimates of the corrected Akaike information criterion (AIC) that can be used to determine the best model. Dominance analyses were subsequently conducted using the R package *dominanceanalysis* (Navarrete & Soares, 2020).

### Results and discussion

**Fig. 3:**
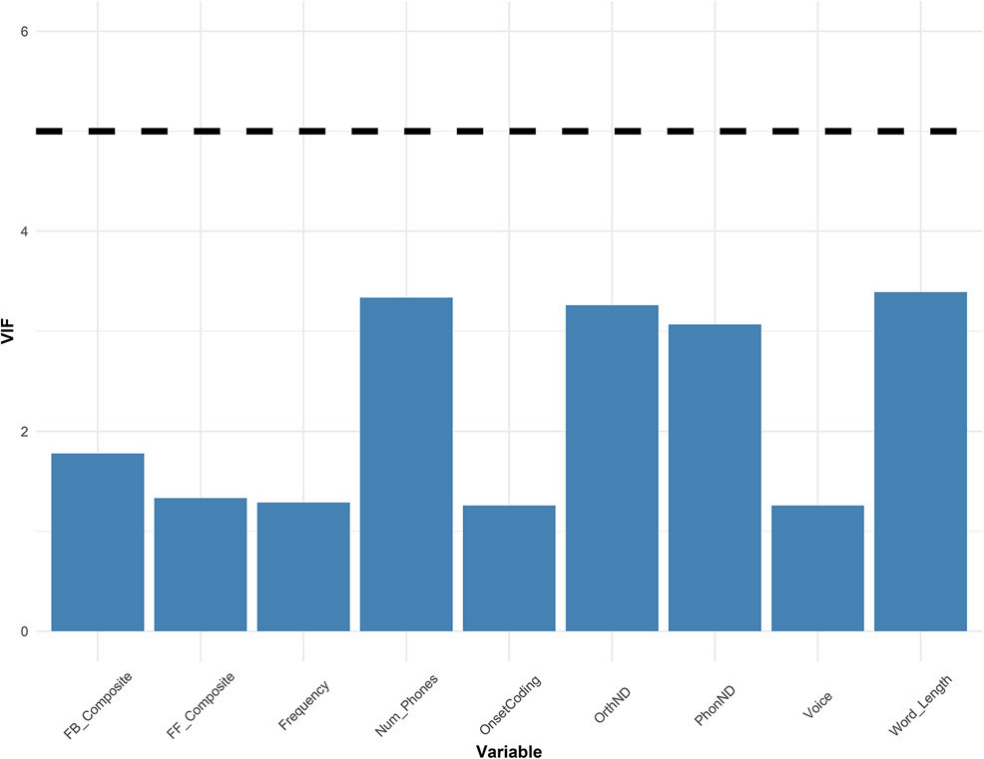
VIF values of all predictors in the ELP dataset, where the accepted threshold is set at < 5

The best-fitting model was found to be the one containing the composite predictor FB_Composite, providing the lowest AIC value (an established information-theoretic measure of model complexity) (Table 3). This finding suggests that expert readers utilize phoneme-to-grapheme consistency information to achieve fluent word reading, corroborating the feedback consistency effects found in previous word naming studies (Balota et al., 2004; Yap & Balota, 2009). Prior to regression analysis, we tested for multicollinearity in the independent variables with the VIF statistic and found no issues (Fig. 3). Generally, a VIF larger than 5 suggests moderate influence, and a value larger than 10 is seen as a strong indicator of multicollinearity (Fox & Weisberg, 2010).

**Table 3:** Comparison of regression models predicting visual naming performance

Model	beta	df	AICc	Delta AICc
FB_Composite	− 0.239	10	10005.42	0.00
FB_PC1	− 0.221	10	10048.44	43.02
FB_Least	− 0.216	10	10056.85	51.43
FB_OVC	− 0.213	10	10058.66	53.24
FB_Rime	− 0.154	10	10147.08	141.66
FB_Onset	− 0.128	10	10173.20	167.78
FF_Composite	− 0.124	10	10178.65	173.23
FB_Graph	− 0.143	10	10187.92	182.50
FF_Graph	− 0.109	10	10198.46	193.04
FF_Least	− 0.108	10	10201.67	196.25
FF_PC1	− 0.098	10	10213.21	207.79
FF_OVC	− 0.085	10	10228.17	222.75
FF_Onset	− 0.074	10	10240.72	235.30
FF_Rime	− 0.059	10	10251.23	245.81
Baseline		9	10270.75	265.33

Note—Models are ranked by AICc. For each model, the number of parameter (df) and the Delta AICc are shown. Models with lower AICc values provide better fit

**Fig. 4:**
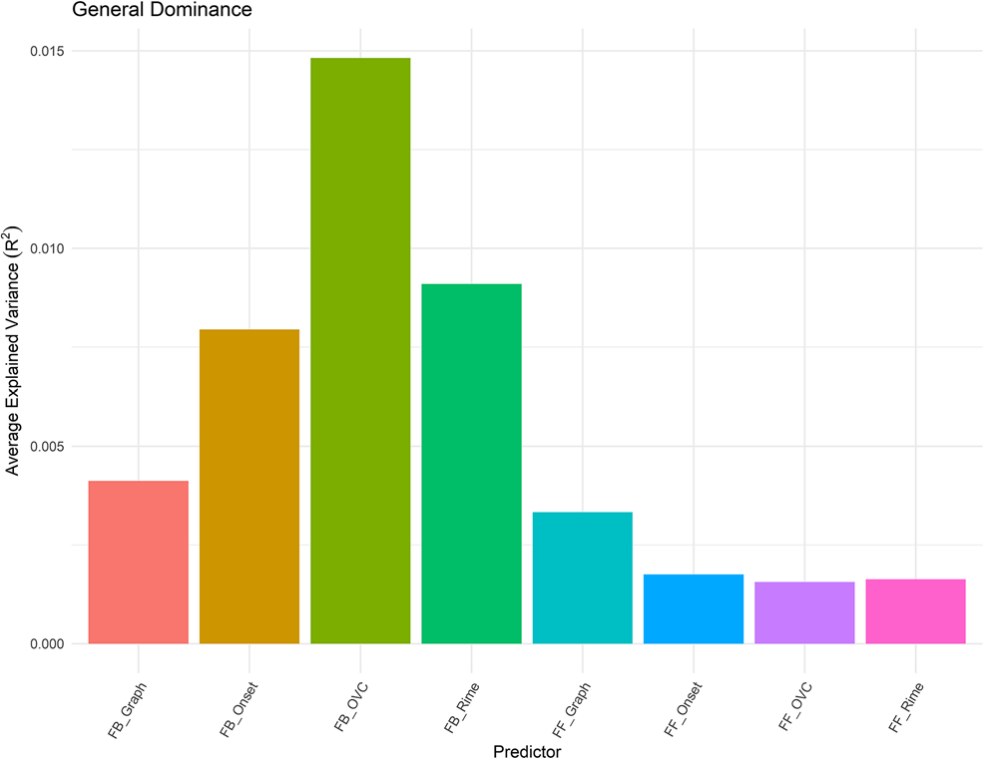
Average variance accounted for in naming task performance by all subset models

The two-step hierarchical analysis revealed that both feedforward and feedback composite scores were good predictors of human naming performance, albeit FB_Composite explained more variance in the data than FF_Composite (Table 4). This suggests that both feedforward and feedback consistency effects are present while reading words out loud. In the subsequent regression models we compared the consistency measures across granularity with the composite measures, because the 14 models were the same but for one predictor (one of the 14 measures of consistency we derived from the corpus) allowing us to rank the models. Further dominance analysis showed that the consistency measure derived at the OVC and grapheme level contributed the most to both the feedback and feedforward consistency effects observed, respectively (Fig. 4).

**Table 4:** Results of hierarchical regression analyses for visual naming task performance

Predictor	*beta*	*beta* 95% CI	Fit
Step 1			
Frequency	− 0.30**	[− 0.33, − 0.28]	
Voice	− 0.26**	[− 0.29, − 0.23]	
Onset_Coding	0.08**	[0.05, 0.10]	
Word_Length	0.28**	[0.24, 0.33]	
Num_Phones	− 0.16**	[− 0.20, − 0.12]	
OrthND	− 0.11**	[− 0.15, − 0.06]	
PhonND	0.04	[− 0.01, 0.08]	
			*R*^2^ =.330** 95% CI[.31, .35]
Step 2			
FB_Composite	− 0.22**	[− 0.25,− 0.19]	
FF_Composite	− 0.03*	[− 0.06, − 0.01]	
			*R*^2^ =.372** 95% CI[.35, .39] Δ *R*^2^ =.042** 95% CI[.03, .05]

Note—*beta* indicates the standardized regression weights. CI indicates the lower and upper limits of a confidence interval, respectively. * indicates *p* < .05. ** indicates *p* < .01

The finding of a feedforward consistency effect is not surprising as extensive findings have shown that spelling-to-sound correspondence plays a role in naming task performance (e.g., Hino & Lupker, 1998; Monsell, Doyle, & Haggard, 1989; Plaut, McClelland, Seidenberg, & Patterson, 1996; Seidenberg, 1992; Van Orden, Pennington, & Stone, 1990). It is notable that the composite measure of feedforward consistency explained more variance in word naming than any of the unit-level feedforward consistency measures, suggesting that these previously used metrics each capture human performance only partially. Feedback consistency effects, on the other hand, have been less systematically observed across studies. Studies have sometimes failed to replicate feedback consistency effects on naming latencies (e.g., Massaro & Jesse, 2005; Peereman, Content, & Bonin, 1998), which were likely due to uncontrolled variables. In a more recent megastudy, Cortese, Yates, Schock, and Vilks (2018), after controlling for surface and lexical variables, found a feedback consistency effect in naming but not lexical decision tasks. Their findings suggest that semantic information has a more critical role in generating lexical decision outputs than the phonological code. In tasks that rely on orthographic-to-phonological decoding such as naming, initial orthographic inputs can trigger a resonance effect from the phonological-to-orthographic levels as a result of interactive activation, causing interference at the orthographic level for feedback inconsistent words. Our results further demonstrated that feedback consistency has a reliable effect on human naming performance, and it has the strongest effect when derived at the OVC level, followed by rime and onset level. In terms of magnitude, it is worth noting that Cortese et al., (2018) also found a stronger rime- than onset-consistency effect in the feedback direction, similar to the one observed in our analyses.

Although many previous studies have found evidence that reading aloud involves phonological processing, it is mostly found for low-frequency words, which does not explain why feedback consistency had a stronger effect than feedforward consistency in our present study. In line with the bi-modal interactive activation model (Frost and Katz, 1989, BIAM;) that was initially designed to account for automatic involvement of phonological information during visual word recognition, one explanation would be that initial orthographic inputs activate phonological representations, which in turn influence the course of visual word recognition via their interaction with orthographic representations.

## Study 2: Consistency effects on lexical decision across modalities

While both word naming and lexical decision involve lexical access and word recognition, lexical decision tasks (LDT) do not overtly require phonological articulation. As such, it is informative to consider whether word consistency impacts mainly the lexical access phase, or the phonological output phases of word processing. Thus, in Study 2 the same consistency measures derived in Study 1 are used here to predict lexical decision performance. Comparing the results to that of Study 1 will enlighten the processing that is most impacted by word consistency.

In fact, while feedforward consistency plays a role in naming task performance, its role in lexical decision has been less well-defined, with the majority of findings suggesting that feedforward consistency has no effect on lexical decision (e.g., Hino & Lupker, 1996; Stanovich & Bauer, 1978; Waters & Seidenberg, 1985), except when phonological processing is emphasized by the task. More recently, however, when feedforward consistency was measured at the onset level, its effects were observed in both naming (e.g., Yap & Balota, 2009; Cortese & Schock, 2013) and lexical decision (e.g., Yap & Balota, 2009; Balota et al., 2004), albeit less consistently and more weakly than when measured at the rime level. These recent results suggest that consistency operationalized at different granularities can lead to different prediction outcomes.

Secondly, following many previous findings that the consistency of printed words holds cross-modal effects, we also compare lexical decision performance in visual formats (judgements of printed words and pseudowords) with auditory formats (judgements of spoken words and pseudowords). As consistency has been reported to affect auditory lexical decision (Pattamadilok, Morais, Ventura, & Kolinsky, 2007; Ventura et al., 2004; Petrova, Gaskell, & Ferrand, 2011; Ventura, Morais, & Kolinsky, 2007; Ventura et al., 2004), we further examine whether such effects are isolated to feedback consistency (sound-to-spelling), which we expect given the sound-based input of the task.

To compare differential effects of directional consistency (feedforward vs feedback) on different modalities of word recognition (visual, auditory) we use our combined consistency metrics in each direction to predict the ELP *visual* lexical decision times on the one hand, and MALD *auditory* lexical decision times on the other. As noted above, we predicted that our feedforward consistency measure would explain the most variance in visual LDT, as found in previous studies (Kessler, Treiman, & Mullennix, 2007), whereas feedback consistency would explain most variance in the auditory LDT following reliable effects reported across studies (e.g., Chng et al., 2019).

### Procedure

After excluding incorrect trials ($\sim 8.84\%$ of all trials), “too fast” responses ($\sim 0.36\%$), and slow outliers ($\sim 6.70\%$), statistical analyses were performed on the remaining $\sim 84.09\%$ of trials. Item-level regression analyses were conducted on the mean *z*-scored RTs for 4207 monosyllabic words for the visual lexical decision task that were obtained from the ELP.

### Results and discussion

**Fig. 5:**
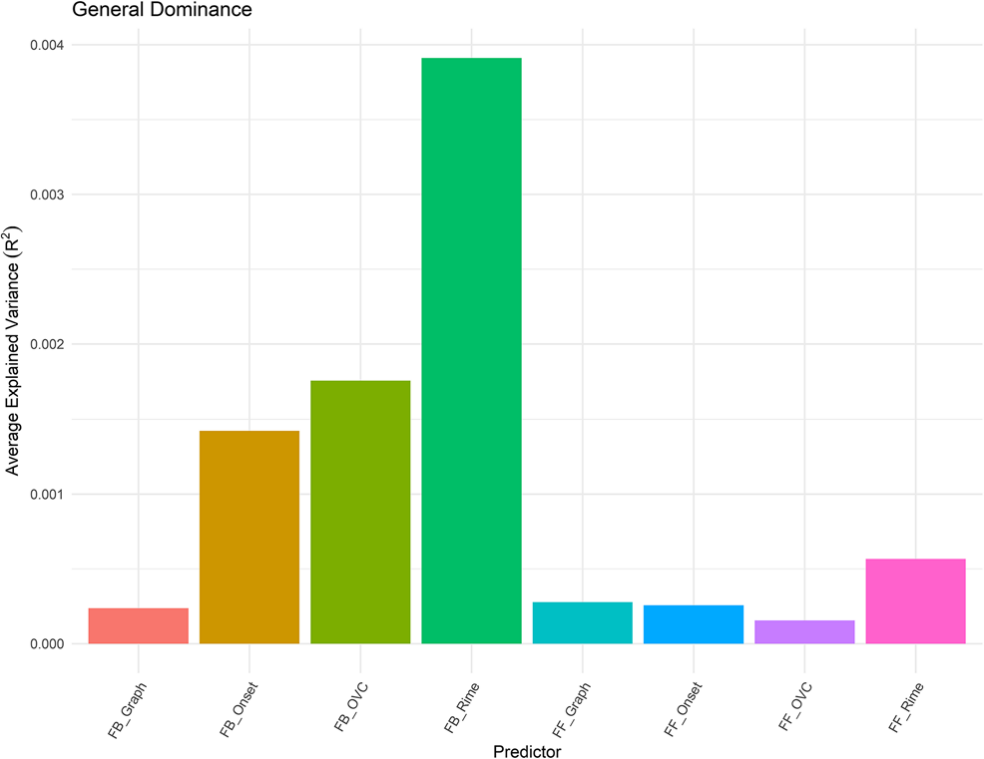
Average variance accounted for in visual lexical decision task performance by all subset models

Entering each consistency measure one-by-one into the individual regression models, we found a similar pattern as previous results with FF_Composite and FB_Composite models performed the best among models in the same direction (Table 5). When both feedforward and feedback composite consistency measures entered the regression model in a two-step hierarchical analysis, only the feedback composite score was significant, with feedback consistent items producing faster latencies (*beta* = − 0.09, 95% CI[− 0.12,− 0.06]) (Table 6). After controlling for lexical variables, adding feedback consistency still resulted in a small but significant increase in the variance accounted for (*Δ* *R*^2^ =.006**). Finally, unlike in Study 1, our dominance analysis revealed that FB_Rime contributed the most to the feedback composite score, followed by FB_OVC and FB_Onset (Fig. 5).

**Table 5:** Comparison of regression models predicting visual lexical decision performance

Model	beta	df	AICc	Delta AICc
FB_Composite	− 0.093	10	9950.91	0.00
FB_Rime	− 0.086	10	9952.08	1.16
FB_Least	− 0.088	10	9955.90	4.99
FB_PC1	− 0.079	10	9963.19	12.28
FB_OVC	− 0.076	10	9964.74	13.82
FB_Onset	− 0.045	10	9980.20	29.29
FF_Composite	− 0.040	10	9983.08	32.17
FF_Graph	− 0.032	10	9986.36	35.44
FF_Rime	− 0.032	10	9986.39	35.48
FF_Least	− 0.031	10	9986.94	36.03
FB_Graph	− 0.038	10	9986.96	36.05
FF_PC1	− 0.031	10	9987.01	36.10
FF_OVC	− 0.027	10	9988.55	37.63
Baseline		9	9991.33	40.42
FF_Onset	− 0.012	10	9992.39	41.48

Note—Models are ranked by AICc. For each model, the number of parameter (df) and the Delta AICc are shown. Models with lower AICc values provide better fit

**Table 6:** Results of hierarchical regression analyses for visual lexical decision task performance

Predictor	*beta*	*beta* 95% CI	Fit
Step 1			
Frequency	− 0.64**	[− 0.66, − 0.61]	
Voice	− 0.01	[− 0.04, 0.01]	
Onset_Coding	0.01	[− 0.02, 0.03]	
Word_Length	0.07**	[ 0.03, 0.11]	
Num_Phones	− 0.20**	[− 0.24, − 0.16]	
OrthND	0.03	[− 0.01, 0.07]	
PhonND	− 0.05*	[− 0.09, -0.01]	
			*R*^2^ =.374** 95% CI[.35, .39]
Step 2			
FB_Composite	− 0.09**	[− 0.12, − 0.06]	
FF_Composite	0.00	[− 0.03, 0.03]	
			*R*^2^ =.380** 95% CI[.36, .40] *Δ* *R*^2^ =.006** 95% CI[.00, .01]

Note—*beta* indicates the standardized regression weights. CI indicates the lower and upper limits of a confidence interval, respectively. * indicates *p* < .05. ** indicates *p* < .01

Previous equivocal findings have suggested that feedback consistency influences naming but not lexical decision (e.g., Balota et al., 2004; Cortese et al., 2018; Yap & Balota, 2009), while others have found its effects in lexical decision (e.g., Lacruz & Folk, 2004; Perry, 2003; Stone et al., 1997). This discrepancy of feedback consistency results may be due to that studies have used different subsyllabic units to calculate consistency. The present study compared feedback consistency measured at different granularity levels and found supporting evidence that the rime-level consistency effects are stronger than that measured at the onset level. When measured at a smaller granularity level, FB_Graph (i.e., feedback grapheme-level consistency) accounted for much less average variance than FB_Onset, as shown in the results of the dominance analysis. This is perhaps due to English readers becoming attuned at a young age to within-word contexts that disambiguate the small-scale grapheme–phoneme inconsistencies (which abound) in favor of larger scale spelling-to-sound correspondences that provide greater consistency (Treiman et al., 1995). Our results thus suggest that consistency effects have to be examined by taking grain sizes into account.

Taken together with previous findings of feedback consistency effects in similar tasks (e.g., Lacruz & Folk, 2004; Perry, 2003; Stone et al., 1997), it is possible that visual lexical decision relies on both phonological and semantic information. In terms of the triangle model of reading (from parallel distributed processing, PDP, neural network models), the process of making a lexical decision may involve orthographic-to-semantic and phonological-to semantic connections. However, because the relationships between orthography and semantics are more arbitrary than those between orthography and phonology (see for a discussion of writing systems ; Frost, 2005), the activated phonological representations by orthographic input would also serve as an input to the semantic system, forming an orthographic-phonological-semantics interaction. Similar to when performing a naming task, the activation of the phonological code would, in turn, either facilitate or interfere with the orthographic representations depending the word’s feedback consistency. This orthographic-phonological-orthographic resonance effect is thought to be less profound in lexical decision tasks, probably due to the lexical decision being made on the basis of semantic information unlike a naming response that is driven by phonological information. This is demonstrated in the two-step hierarchical regression results of studies 1 (naming) and 2 (lexical decision) where the composite consistency measures contributed more unique variance in the former task (i.e., an increased *R*^2^ by 0.042 vs. 0.006, respectively).

### Predicting auditory lexical processing in the MALD dataset

#### Procedure

**Fig. 6:**
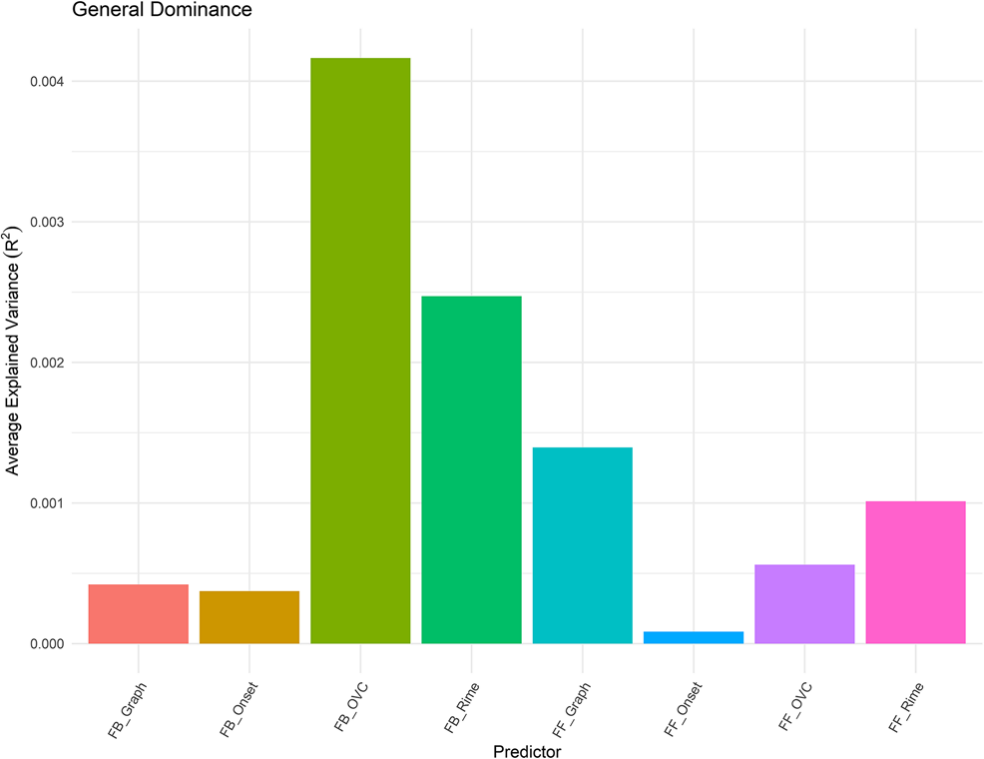
Average variance accounted for in auditory lexical decision task performance by all subset models

**Fig. 7:**
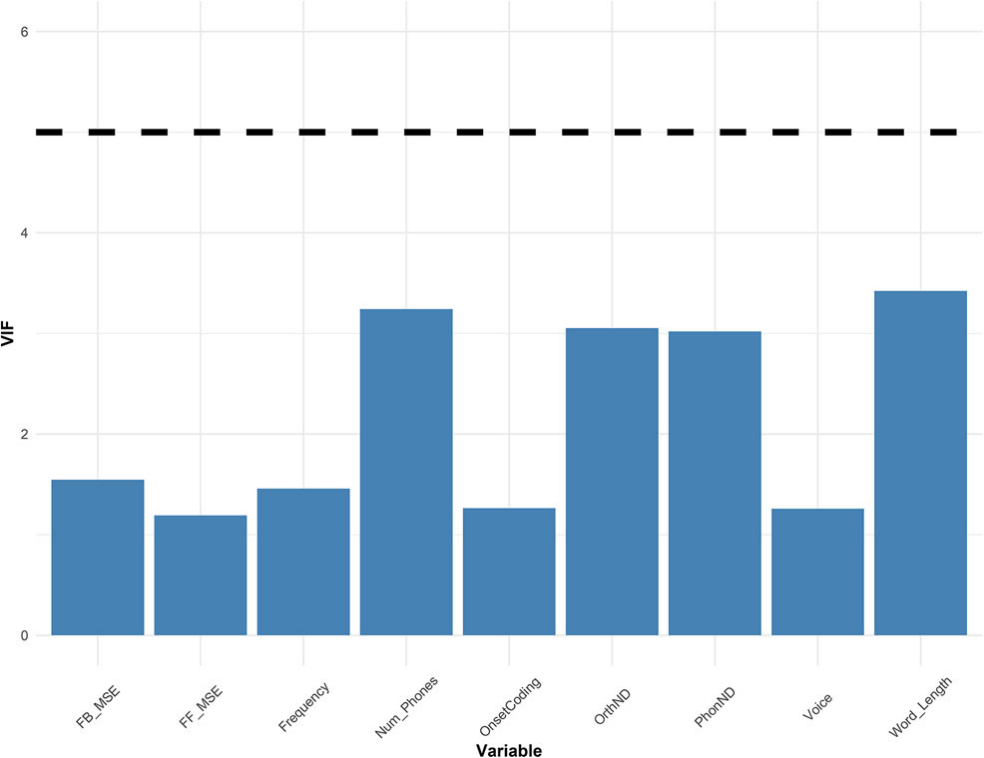
VIF values of all predictors in the MALD dataset, where the accepted threshold is set at < 5

After excluding incorrect trials ($\sim 9.18\%$ of all trials), “too fast” responses ($\sim 0.69\%$), and slow outliers ($\sim 6.31\%$), statistical analyses were performed on the remaining $\sim 83.83\%$ of trials. Item-level regression analyses were conducted on the mean *z*-scored RTs for 4341 monosyllabic words for the auditory lexical decision task that were obtained from the MALD (Figs. 6 and 7).

### Predicting visual word recognition from the ELP dataset: Visual lexical decision task

#### Results and discussion

Contrary to the equivocal findings with visual lexical decision tasks discussed earlier, feedback consistency effects have been consistently reported and replicated in the auditory modality of the task (e.g., Ch’ereau, Gaskell, & Dumay, 2007; Miller & Swick, 2003; Pattamadilok et al., 2007; Perre & Ziegler, 2008; Slowiaczek, Soltano, Wieting, & Bishop, 2003; Taft, Castles, Davis, Lazendic, & Nguyen-Hoan, 2008; Ventura et al., 2007; Ziegler et al., 2004; Ziegler & Muneaux, 2007; Ziegler, Muneaux, & Grainger, 2003). It is commonly found that adults are faster and more accurate in auditory lexical decisions tasks for feedback consistent words. In the present study, we too found feedback consistency effects with our composite score (FB_Composite; *beta* = − 0.074, Delta AIC = 6.00) (Table 7). Note that, however, all feedback word-level models have only a small difference in their AIC values, and hence there is a lack of evidence to distinguish the best word-level predictor (FB_PC1 vs. FB_Composite, Delta AIC = 4.11). Among all the consistency measures, FB_OVC (*beta* = − 0.083) was found to be the best predictor of auditory lexical processing, which is likely due to the OVC being the most salient phonological units in English.

**Table 7:** Comparison of regression models predicting auditory lexical decision performance

Model	beta	df	AICc	Delta AICc
FB_OVC	− 0.083	10	12192.93	0.00
FB_PC1	− 0.081	10	12194.82	1.89
FB_Least	− 0.078	10	12196.13	3.20
FB_Rime	− 0.073	10	12196.80	3.87
FB_Composite	− 0.074	10	12198.93	6.00
FF_Rime	− 0.042	10	12209.88	16.95
FF_Least	− 0.038	10	12211.41	18.48
FF_OVC	− 0.031	10	12213.30	20.37
FF_Composite	− 0.028	10	12214.12	21.20
FB_Onset	− 0.029	10	12214.13	21.21
FF_PC1	− 0.027	10	12214.47	21.54
Baseline		9	12215.64	22.71
FF_Onset	− 0.015	10	12216.75	23.83
FF_Graph	0.012	10	12216.97	24.04
FB_Graph	− 0.010	10	12217.37	24.44

Note—Models are ranked by AICc. For each model, the number of parameter (df) and the Delta AICc are shown. Models with lower AICc values provide better fit

In a developmental study by Ziegler and Muneaux (2007), they showed that auditory lexical decision performance was not initially influenced by feedback consistency, however, as soon as literacy developed, feedback consistency effects were observed with its magnitude predictable by the reading level of the child. In terms of neural network models of reading, this implies that the processing of visual and spoken words is tightly linked through a single network that connects both the orthographic and phonological layers. Thus, in order for the network to process a spoken word via phonological code activation, the corresponding orthographic code has to be coactivated as well, due to the strong orthographic-phonological associations.

Perre and Ziegler (2008) explained that the permanent orthographic-phonological connections are likely formed during literacy learning, and competition occurs at the orthographic layer when a word has multiple spellings (i.e., feedback inconsistent words). However, because the mapping between orthographic sub-units and semantic features is less systematic, phonology plays a more important role in accessing word meaning (e.g., Amenta, Marelli, & Sulpizio, 2017; Tyler, Voice, & Moss, 2000). When participants were presented with homophones and non-homophonic words in a lexical task, it is typically found that responses for homophones are slower as compared to non-homophonic words (e.g., Ferrand & Grainger, 2003; Pexman, Lupker, & Jared, 2001; Besner & Davelaar, 1983; Coltheart, Davelaar, Jonasson, & Besner, 1977; McCann, Besner, & Davelaar, 1988; Mcquade, 1981; Vanhoy & Van Orden, 2001; Ziegler, Jacobs, & Kluppel, 2001; Rubenstein, Lewis, & Rubenstein, 1971), which further suggests that phonological recoding of a printed word plays an important role in word recognition.

We also note that across the different datasets that were modeled, the regression model for auditory lexical decision accounted for a relatively modest amount of variance even with the inclusion of the composite consistency measures (*R*^2^ = .032) (Table 8). This could be due to the lack of semantic variables in the model, as these have been found to account for more incremental variance in lexical decision, and which is consistent with lexical decision’s emphasis on semantic information.

**Table 8:** Results of hierarchical regression analyses for auditory lexical decision task performance

Predictor	*beta*	*beta* 95% CI	Fit
Step 1			
Frequency	− 0.02	[− 0.05, 0.01]	
Voice	− 0.11**	[− 0.15, − 0.08]	
Onset_Coding	− 0.04*	[− 0.07, − 0.01]	
Word_Length	0.00	[− 0.06, 0.05]	
Num_Phones	0.14**	[0.09, 0.19]	
OrthND	− 0.06*	[− 0.12, − 0.01]	
PhonND	0.13**	[0.08, 0.17]	
			*R*^2^ =.027** 95% CI[.02, .04]
Step 2			
FB_Composite	− 0.08**	[− 0.11, -0.04]	
FF_Composite	0.00	[− 0.03, 0.04]	
			*R*^2^ =.032** 95% CI[.02, .04] *Δ* *R*^2^ =.004** 95% CI[.00, .01]

Note—*beta* indicates the standardized regression weights. CI indicates the lower and upper limits of a confidence interval, respectively. * indicates *p* < .05. ** indicates *p* < .01

## Study 3: Data-driven measures of consistency

Systematic resonance between orthographic and phonological units in reading has been observed and put forward previously (e.g., Frost & Katz, 1989; McClelland & Rumelhart, 1981; Stone & Van Orden, 1994; Van Orden & Goldinger, 1994), suggesting that information does not flow in only one direction. In an explicitly interactive model of reading, words that are consistent in both feedforward and feedback directions guarantee stabler and faster learning, which also leads to fast activation due to consistent symmetrical relations that can be resolved more quickly as compared to asymmetrical ones—i.e., words that are consistent only in one direction but not the other (Tuller, Case, Ding, & Kelso, 1994; Van Orden, 2002; Van Orden, Jansen op Haar, & de Bosman, 1997; Van Orden, Pennington, & Stone, 1990; Ziegler, Van Orden, & Jacobs, 1997c).

Our findings thus far are consistent with such an interactive account. First, in Study 1, we found both feedforward and feedback consistency effects in a visual naming task, supporting the notion that phonology is involved in visual word recognition, and both inconsistent orthography-phonology and phonology-orthography mappings can slow the process of visual word recognition. Second, in Study 2, we found feedback consistency effects in both visual and auditory lexical decision tasks, implying that feedback consistency plays a role in not only reading but also in spoken word recognition. Taken together, the role of phonological computation appears crucial for print processing and lexical access (see for a review ; Frost, 1998), and this is likely due to orthographic-phonological resonance and phonological information being the primary mechanism by which we retrieve meaning. Thus, the findings offer a demonstration that the orthographic and phonological systems are closely interconnected and the flow of information is bidirectional, regardless of whether the input is visual or auditory.

In Study 3, we aimed to further validate the bidirectional interaction hypothesis between orthographic and phonological systems by modelling it explicitly in a computational neural network that learned to read words. We expected that consistency effects are detectable in the learning process of a reading/writing model, and emerge from statistical regularities present in the language, in particular, the correspondence between words’ orthographic and phonological forms. To emulate this process, we employed a machine learning regime and derived a proxy for the difficulty of learning each word in our corpus.

A neural network model was trained with either an orthography-phonology or phonology-orthography mapping task, corresponding to reading aloud visually presented words, and spelling spoken words, respectively. Our focus is on the PDP framework developed by Rumelhart, Hinton, and McClelland (1986) that provides natural accounts of the exploitation of multiple, simultaneous, and often mutual constraints. To examine the ease with which the model can generate the target output for a word, we measured the closeness of the model’s output to the target by calculating the mean squared error (MSE) that serves as a reflection of how difficult it was for the model to learn the GPC/PGC mappings of each word.

Researchers have used also MSE as a measure of response time in PDP models (e.g., Seidenberg & McClelland, 1989; Monaghan & Pollmann, 2003), but this approach has since been supplemented by response time measures, such as the amount of continuous time needed for output unit activations to settle (e.g., Monaghan, Shillcock, & McDonald, 2004; Zorzi, Houghton, & Butterworth, 1998; Seidenberg & Plaut, 1998).

MSE is an ideal proxy measure for spelling-sound consistency because of its link to the concept of cross-entropy from information theory (Kullback & Leibler, 1951), which measures the similarity of two probability distributions. Since our goal of modelling is to identify words with different levels of spelling-sound and sound-spelling consistency, then the cross-entropy of consistent words is expected to be lower than that of inconsistent words, as the model can minimize the cross-entropy of consistent patterns faster (i.e., in a lesser number of training epochs) than inconsistent patterns (e.g., Plaut et al., 1996). Here, we expect relatively fast and stable responses for consistent compared to inconsistent words, and, therefore, consistent words should exhibit a lower MSE as compared to inconsistent words.

At completion of network training, such MSE measures of individual word consistency were then entered as an independent variable in linear models predicting ELP visual LDT and MALD auditory LDT. Finally, we compared the linear models containing the data-driven neural network predictors with the linear models containing the corpus-derived metrics of consistency, and ascertained which models fit the human data best.

### Model architecture

The model’s architecture is most similar to the connectionist triangle model of Harm and Seidenberg (2004) with the addition of an orthographic attractor to encode information about the orthographic structure of English, as well as bidirectional connections between layers. A semantic layer, which is part of the original triangle model, was not included, as our task was to assess spelling-to-phonology and phonology-to-spelling consistency.

**Fig. 8:**
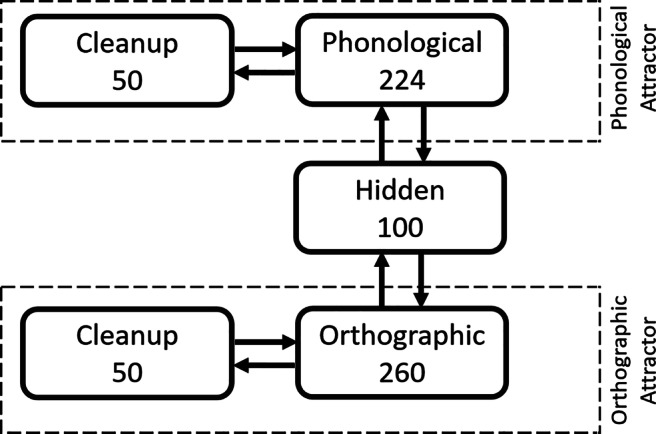
Architecture of the reading and spelling connectionist model implemented in Study 3

The model was built using the free software LENS (Rohde, 1999) and has four types of layers: orthographic, phonological, hidden, and clean-up/attractor units (see Fig. 8). The hidden layer mediated the computations between orthographic and phonological codes, allowing the network to encode more complex and latent mappings. In addition, the orthographic and phonological layers were each connected to clean-up layers, creating attractor networks that could settle into a stable pattern over training (Harm & Seidenberg, 1999). All connections between the connected layers were bidirectional.

An attractor network can repair partial or degraded patterns of activity by pulling nearby points toward the stable attractor points, and by turning noisy patterns to familiar representations (Harm & Seidenberg, 1999). The purpose of introducing clean-up units to the orthographic and phonological layers is so that the network can encode orthographic and phonological regularities. With it, the connections between orthographic and phonological layers can be less precise as the model can rely on the attractors to complete the pattern (Harm & Seidenberg, 2004). Some connectionist reading models trade off model stability for a higher sensitivity to new inputs (Hebb, 1949), by foregoing the attractor algorithm and clean-up units (e.g., Lambon Ralph & Ehsan, 2006; Ellis & Lambon Ralph, 2000). We opted to emphasize model stability, following similar connectionist models for reading.

We used a position sensitive slot-based vowel-centered format for both orthographic and phonological representation (e.g., Harm & Seidenberg, 1999; 2004). The orthographic layer was composed of 260 units, corresponding to ten letter position slots × 26 possible letters. Words were coded as vowel-centered, such that the fourth slot was filled with the left-most vowel of a word (e.g., *mince* → *_ _ m i n c e _ _ _*, (e.g., Harm & Seidenberg, 2004; Monaghan, Chang, Welbourne, & Brysbaert, 2017). A word’s phonology was represented with nodes coding phoneme features (eight phoneme position slots × 28 possible phonological features = 224 units). Each phoneme was encoded by a binary vector of 28 phonological features (e.g., anterior, approximant, back, consonantal, etc.) taken from PHOIBLE (Moran & McCloy, 2019), an online repository of cross-lingual phonological data. The value of 1 represented the presence of that feature and 0 represented its absence. A list of phonemes and their respective phonological features used in the present work can be found in the Open Science Framework (OSF) repository for this project (https://osf.io/wdzqc). Full documentation of the model architecture and source code can be found in the GitHub repository (https://github.com/alfred-lim/BiPDP).

### Training procedure

The network was trained to learn the mappings in either one of the two directions, print-to-sound (reading task) or sound-to-print (spelling task). Training was done separately and exclusively in one direction because we wanted to ensure that the two effects were not confounded, as may occur with interleaved training. In addition, each of the reading and spelling models was trained using two measures of word-frequency: one based on type frequencies and the other based on token frequency, resulting in a total of four models being trained. We reasoned that the different frequency-weighted training approaches would produce MSEs that are analogous to token and type consistencies derived from a corpus.

When a phonological word was presented to the network’s phonological layer (e.g., to simulate a word spelling task), its activation would spread to the hidden layer, followed by the orthographic layer. Conversely, in the reading task, an orthographic word would be input to the orthographic layer, and its activation cascaded to the phonological layer via the same hidden units. Bidirectional connections between orthographic-hidden-phonological layers provide an opportunity for the output layer to influence the rise of activation of units in the input layer. For example, when the word PINT is presented to the network in the reading task, the orthographic nodes for PINT will spread its activity to the corresponding hidden nodes, and then to the phonological nodes through feedforward activation. However, the orthographic nodes for PINT will also receive activation from phonological nodes via the hidden layer as a result of feedback connections, simulating the resonance effect described in the previous studies.

All models were trained with a learning rate of 0.05 using a back-propagation through time (BPTT) algorithm (Harm & Seidenberg, 1999; Plaut, McClelland, Seidenberg, & Patterson, 1996) with input integration and a time constant of 0.5. The weight connections were updated based on cross-entropy error computed between the target and the actual activation of the output units.

The input pattern of each word in the corpus was clamped and presented for six time samples, then in an additional six time samples the model was required to reproduce the target pattern of the word. Both the orthography-to-phonology and phonology-to-orthography target mappings were taken from the MALD corpus. A node was considered activated if its output was greater than 0.75 and deactivated if less than 0.25, while intermediate values were considered incorrect. In other words, an output was scored as correct when the target nodes were active with a value >= 0.75, and concurrently the other nodes were inactive (<= 0.25).

### Results and discussion

The goal of modeling was to inspect the relevance of using the model’s MSEs as a measure of consistency, which we referred to as data-*driven* consistency (in contrast to the corpus-*derived* measures of Study 1 and 2). As we are interested in capturing the relative ’ease of learning’ for each word in terms of MSE, the models were trained until performance reached a reasonable plateau for all tasks and training regimes to avoid over-fitting. Further, we used a cut-off point of 100,000 epochs as a stopping criteria instead of an accuracy criteria, in order to prevent lower accuracy models from having more exposure to the stimuli.

**Fig. 9:**
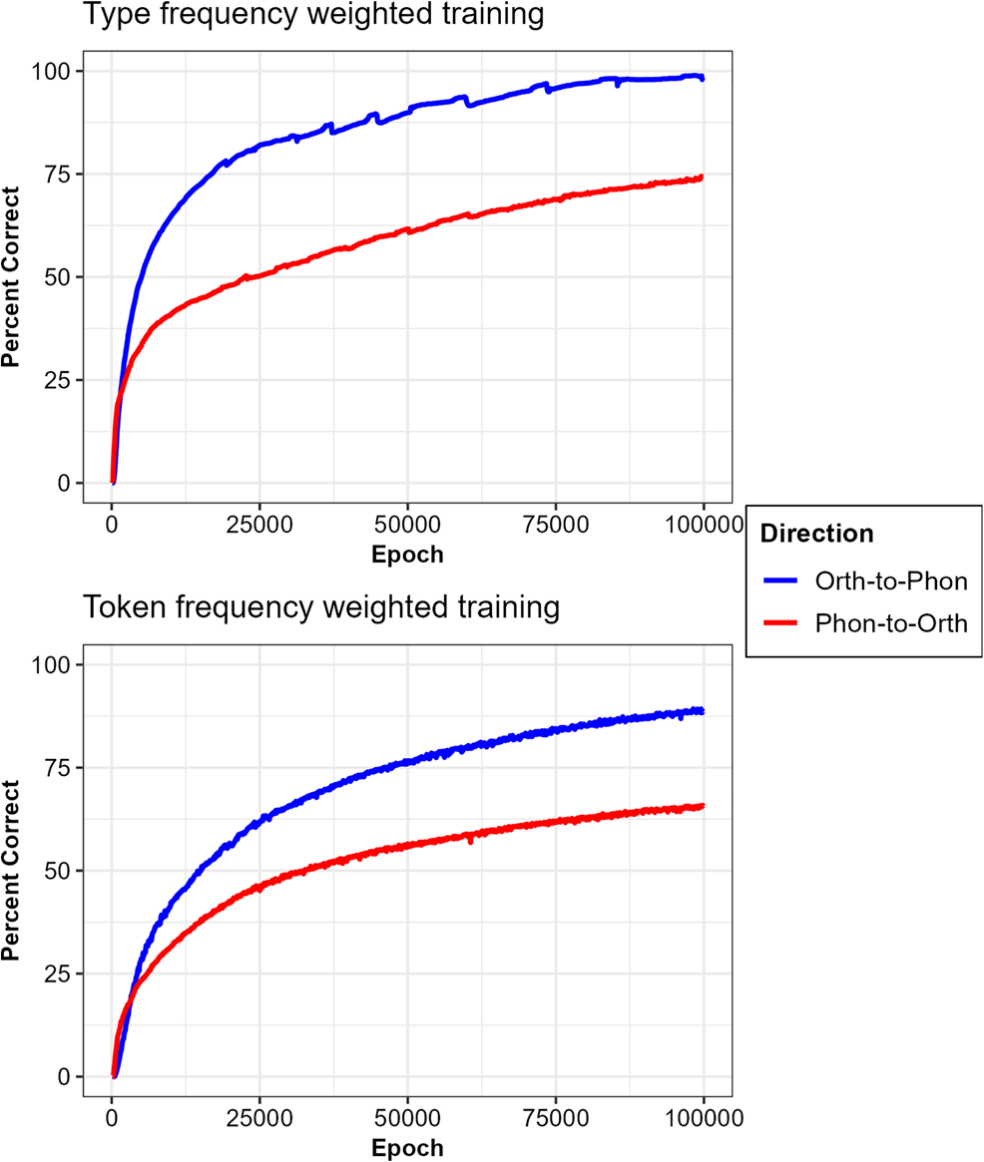
Network accuracy scores on the reading (orthographic-to-phonology) and spelling (phonology-to-orthographic) tasks that were trained using either type or token word frequency

Accuracy over the course of the training is depicted in Fig. 9. Both the reading and spelling models trained using type frequency showed higher levels of accuracy at the end of training (98.9 and 74.3% correct words, respectively) as compared to those trained using token frequency (89.1 and 65.9% correct words). This is likely due to all words having the same chance of being presented to the model in type-frequency training wherein the network was able to better capture regularities among inputs as compared to the token-frequency training. Also, the models were able to learn the orthography-to-phonology mappings better than phonology-to-orthography, likely as a result of there being many more ways to spell a given phoneme in English than there are different ways of pronouncing a particular grapheme (e.g., Goswami & Bryant, 1990).

To examine the impact of word consistency on token-weighted MSEs in the two tasks, we divided the words into two equal-sized groups based upon either their feedforward or feedback composite scores using median as a cut-off value: inconsistent (*N* = 2173) and consistent (*N* = 2173). When compared using MSEs derived from the same direction as the nature of the task, reading (i.e., feedforward) MSEs were higher for words that are feedforward-inconsistent [*M* = .0039,*SD* = .0082] than feedforward-consistent [*M* = .0019,*SD* = .0043; *t*(4344) = 9.75,*p* < .001], and spelling (i.e., feedback) MSEs were worse for feedback-inconsistent [*M* = .0047,*SD* = .0048] than feedback-consistent words [*M* = .0015,*SD* = .0025; *t*(4344) = 27.47,*p* < .001], indicating that the models were able to capture consistency effects in both directions.

To further validate if the computed data-driven MSE is appropriate as a proxy measure of print-speech consistency, we conducted a new set of regression analyses on the three sets of human performance data with the addition of feedforward (FF_MSE) and feedback MSE (FB_MSE). To include a parsimonious set of predictors in these models, only the previous best individual consistency measures (i.e., OVC) and the composite consistency measures (FF_Composite and FB_Composite) were compared.

#### Predicting visual naming latencies in the ELP dataset

After controlling for lexical variables, model selection analysis based on AIC revealed that all single-predictor models performed better than the baseline (Table 9). Even though the models trained with type frequency resulted in higher accuracy of neural network performance than token frequency training overall, the obtained MSE-consistency measures from the token frequency training arose as better predictors of human word naming (FF_MSE, AIC = 10117; FB_MSE, AIC = 10130) as compared to type frequency-training (FF_Type_MSE, AIC = 10155; FB_Type_MSE, AIC = 10211). This dovetails with previous findings whereby consistency weighted by token frequency is more predictive of human performance in naming tasks as compared to type frequency (Jared, McRae, & Seidenberg, 1990; Lee, Tsai, Su, Tzeng, & Hung, 2005). Furthermore, similar to the composite consistency effects observed in Study 1, the feedback MSE model had a lower AIC than its feedforward counterpart. However, the previous FB_Composite model from Study 1 still performed better than the MSE model in predicting visual word naming.

**Table 9:** Comparison of regression models predicting visual naming performance

Model	beta	df	AICc	Delta AICc
FB_Composite	− 0.239	10	10005.42	0.00
FF_MSE	0.163	10	10116.76	111.34
FB_MSE	0.177	10	10130.47	125.05
FF_Type_MSE	0.137	10	10155.46	150.04
FF_Composite	− 0.124	10	10178.65	173.23
FB_Type_MSE	0.115	10	10211.03	205.60
Baseline		9	10270.75	265.33

Note—Models are ranked by AICc. For each model, the number of parameter (df) and the Delta AICc are shown. Models with lower AICc values provide better fit

To determine if the new data-driven consistency measures accounted for additional variance in the word naming RTs over conventional lexical variables and the corpus-derived composite measures, we conducted a three-step regression analysis where lexical variables were entered into the regression model in Step 1, followed by composite consistency measures (FB_Composite, FF_Composite) in Step 2, and finally the data-driven consistency measures (FB_MSE, FF_MSE) in Step 3. The final model significantly predicted naming latencies, accounting for 39% of the variance (*R*^2^ = .387, 95% CI[.36,.41]). As seen in Table 10, apart from the control variables, the final model contained three statistically significant predictors: feedback composite, feedback MSE, and feedforward MSE. The addition of MSEs contributed significant improvement in the model (*ΔR*^2^ =.015, 95% CI[.01, .02]).

**Table 10:** Results of hierarchical regression analyses for visual naming task performance

Predictor	*beta*	*beta* 95% CI	Fit
Step 1			
Frequency	− 0.30**	[− 0.33, − 0.28]	
Voice	− 0.26**	[− 0.29, − 0.23]	
Onset_Coding	0.08**	[ 0.05, 0.10]	
Word_Length	0.28**	[ 0.24, 0.33]	
Num_Phones	− 0.16**	[− 0.20, − 0.12]	
OrthND	− 0.11**	[− 0.15, − 0.06]	
PhonND	0.04	[− 0.01, 0.08]	
			*R*^2^ =.330** 95% CI[.31, .35]
Step 2			
FB_Composite	− 0.22**	[− 0.25, − 0.19]	
FF_Composite	− 0.03*	[− 0.06, − 0.01]	
			*R*^2^ =.372** 95% CI[.35, .39] *Δ* *R*^2^ =.042** 95% CI[.03, .05]
Step 3			
FB_MSE	0.07**	[0.04, 0.10]	
FF_MSE	0.11**	[0.08, 0.13]	
			*R*^2^ =.387** 95% CI[.36, .41] *ΔR*^2^ =.015** 95% CI[.01, .02]

Note—*beta* indicates the standardized regression weights. CI indicates the lower and upper limits of a confidence interval, respectively. * indicates *p* < .05. ** indicates *p* < .01

#### Predicting visual lexical processing in the ELP dataset

Similar to the regression models above for visual word naming latency, all single-predictor models performed better than the baseline for visual lexical decision latency (Table 11). Further, MSEs derived from token frequency weighted training were better predictors (FF_MSE, AIC = 9957; FB_MSE, AIC = 9928) than from type frequency weighted training (FF_Type_MSE, AIC = 9964; FB_Type_MSE, AIC = 9982). In each case, feedback MSE also yielded a better model than feedforward MSE. Compared to the corpus-derived consistency measures, the data-driven MSE measures outperformed these in both feedforward and feedback directions. This differs from the prediction of visual word naming reported above, where the corpus-derived feedback consistency measures showed best fit (Fig. 7).

**Table 11:** Comparison of regression models predicting visual lexical decision performance

Model	beta	df	AICc	Delta AICc
FB_MSE	0.116	10	9928.41	0.00
FB_Composite	− 0.093	10	9950.91	22.50
FF_MSE	0.076	10	9957.40	28.99
FF_Type_MSE	0.067	10	9963.82	35.41
FB_Type_MSE	0.047	10	9982.16	53.75
FF_Composite	− 0.040	10	9983.08	54.67
Baseline		9	9991.33	62.92

Note—Models are ranked by AICc. For each model, the number of parameter (df) and the Delta AICc are shown. Models with lower AICc values provide better fit

For the three-step regression analysis of visual lexical decision RTs, the final model accounted for 39% of the variance (*R*^2^ = .387, 95% CI[.36,.41]), and the addition of MSEs contributed significant improvement in the model (*ΔR*^2^ =.007, 95% CI[.00, .01]) (Table 12).

**Table 12:** Results of hierarchical regression analyses for visual lexical decision task performance

Predictor	*beta*	*beta* 95% CI	Fit
Step 1			
Frequency	− 0.64**	[− 0.66, − 0.61]	
Voice	− 0.01	[− 0.04, 0.01]	
Onset_Coding	0.01	[− 0.02, 0.03]	
Word_Length	0.07**	[ 0.03, 0.11]	
Num_Phones	− 0.20**	[− 0.24,− 0.16]	
OrthND	0.03	[− 0.01, 0.07]	
PhonND	− 0.05*	[− 0.09,− 0.01]	
			*R*^2^ =.374** 95% CI[.35, .39]
Step 2			
FB_Composite	− 0.09**	[− 0.12,− 0.06]	
FF_Composite	− 0.00	[− 0.03, 0.03]	
			*R*^2^ =.380** 95% CI[.36, .40] *Δ* *R*^2^ =.006** 95% CI[.00, .01]
Step 3			
FB_MSE	0.08**	[ 0.05, 0.11]	
FF_MSE	0.05**	[ 0.02, 0.07]	
			*R*^2^ =.387** 95% CI[.36, .41] *ΔR*^2^ =.007** 95% CI[.00, .01

Note—*beta* indicates the standardized regression weights. CI indicates the lower and upper limits of a confidence interval, respectively. * indicates *p* < .05. ** indicates *p* < .01

#### Predicting auditory lexical processing in the MALD dataset

Minor differences were observed when comparing the results between auditory and visual lexical decision tasks. First, both the type-weighted MSE models performed worse than their token-weighted counterparts, further supporting that consistency should take token frequency into account (Table 13). Second, FB_MSE (AIC = 12131) is ranked higher than FB_OVC (AIC = 12193, Delta AIC = 61.65) that was found to be the best performing model in Study 2. Lastly, despite that the final three-step model accounting for only a modest 5% of the variance (*R*^2^ = .048, 95% CI[.01,.02]), the addition of MSEs still improved the model significantly (Δ*R*^2^ =.016, 95% CI[.01, .02]) (Table 14). We note that at the second step where composite scores were added to the models, improvement was negligible at a modest 0.4% for auditory lexical decision, lower than that when MSEs were added at the final step.

**Table 13:** Comparison of regression models predicting auditory lexical decision performance

Model	beta	df	AICc	Delta AICc
FB_MSE	0.150	10	12131.28	0.00
FF_MSE	0.084	10	12186.75	55.47
FB_OVC	− 0.083	10	12192.93	61.65
FB_Composite	− 0.074	10	12198.93	67.65
FF_OVC	− 0.031	10	12213.30	82.02
FF_Composite	− 0.028	10	12214.12	82.84
FB_Type_MSE	0.028	10	12215.14	83.86
FF_Type_MSE	0.024	10	12215.22	83.94
Baseline		9	12215.64	84.36

Note—Models are ranked by AICc. For each model, the number of parameter (df) and the Delta AICc are shown. Models with lower AICc values provide better fit

**Table 14:** Results of hierarchical regression analyses for auditory lexical decision task performance

Predictor	*beta*	*beta* 95% CI	Fit
Step 1			
Frequency	− 0.29**	[− 0.33, − 0.26]	
Voice	− 0.13**	[− 0.16, − 0.09]	
Onset_Coding	− 0.05**	[− 0.08, − 0.02]	
Word_Length	− 0.02	[− 0.08, 0.03]	
Num_Phones	0.08**	[0.03, 0.13]	
OrthND	− 0.01	[− 0.06, 0.04]	
PhonND	0.13**	[0.08, 0.18]	
			*R*^2^ =.099** 95% CI[.08, .11]
Step 2			
FB_Composite	− 0.08**	[− 0.11, − 0.04]	
FF_Composite	0.00	[− 0.03, 0.04]	
			*R*^2^ =.032** 95% CI[.02, .04] *Δ* *R*^2^ =.004** 95% CI[.00, .01]
Step 3			
FB_MSE	0.14**	[0.10, 0.17]	
FF_MSE	0.04*	[0.01, 0.07]	
			*R*^2^ =.048** 95% CI[.03, .06] *ΔR*^2^ =.016** 95% CI[.01, .02]

Note—*beta* indicates the standardized regression weights. CI indicates the lower and upper limits of a confidence interval, respectively. * indicates *p* < .05. ** indicates *p* < .01

In sum, across all three data sets, token-weighted consistency measures continued to demonstrate better predictive modeling results as opposed to those that were type-weighted. This is an expected outcome as consistency effects should reflect the influence of statistical patterns across many similar parts of words and, therefore, the most difficult items both in acquisition and processing are those with rare print-sound correspondences that are encountered infrequently (Jared, 2002; Lee et al., 2005). Through the token frequency weighted training using the subtitle-based corpus counts, the connections came to be weighted in such a way that reflects the appropriate relationships between orthography and phonology while taking into account how often readers and listeners encounter a particular type when using the language.

Token-weighted MSEs from both feedforward and feedback directions improved all three-step regression models, albeit to different extents, even when the corpus-derived composite consistency measures have already been included. The most marked improvement was observed in predicting auditory lexical decision performance (1.6*%*), followed by visual naming (1.5*%*), and visual lexical decision (0.7*%*). Although the model improvements contributed by MSEs were the lowest in visual lexical decision among all three tasks, it still contributed explained variance over and above the corpus-derived composite measures. Similar patterns of results were observed across all three tasks, indicating that MSE is a better measure of consistency than the conventional ones in capturing consistency effects in lexical decision tasks.

The auditory lexical decision task is somewhat novel in the word-recognition literature, and findings suggest that the visual and auditory lexical decision tasks are based on different processes (e.g., Rodd, Gaskell, & Marslen-Wilson, 2002; Ernestus & Cutler, 2015; Brysbaert, Stevens, Mandera, & Keuleers, 2016; Segui, 1994; Ferrand et al., 2018). Indeed, our findings of word frequency and length effects in visual lexical decision task are consistent with the results of previous studies. Specifically, faster responses are elicited in visual lexical decision by high-frequency words (e.g., Balota et al., 2007; Brysbaert et al., 2016; Cortese & Khanna, 2007; Keuleers, Lacey, Rastle, & Brysbaert, 2012; Yap & Balota, 2009) and longer words (e.g., New, Ferrand, Pallier, & Brysbaert, 2006; Ferrand et al., 2010; Balota et al., 2007; Brysbaert et al., 2016; Keuleers, Diependaele, & Brysbaert, 2010). These lexical variables do not contribute to response speed in auditory modality lexical decision tasks, however, as seen in our stepwise regression analyses. This same pattern was found by Ferrand et al., (2018) who compared visual and auditory lexical decision times in a megastudy and found that the proportion of variance explained by word frequency is lower in the auditory (11%) than visual (45%) modality. The effect size of word length was also lesser in the auditory as compared to visual modality in their megastudy. Our finding of not only lesser but absent word frequency and length effects on the auditory lexical decision task, we reason, is likely due to the exclusion of multisyllabic words that led to lower statistical power (larger confidence intervals). This is indeed a limitation of the present study, but was necessary as there is no reliable way to compute the different sub-level consistency measures for multisyllabic words without degrading the amount of information that the composite scores provide.

## General discussion

For reading science, the definition of consistency in terms of print-speech mappings is central to theorizing about reading across scripts (the orthographic depth hypothesis; Katz and Feldman, 1983) and reading acquisition (the psycholinguistic grain size theory; Ziegler & Goswami, 2005). In this paper, we defined consistency across different levels or unit sizes (granularity) for the quasi-regular orthography of English, and we compared these different unit-measures in terms of their interrelations, their combination, as well as the ability to predict human oral and silent reading, in addition to auditory word recognition that does not overtly require accessing print information. Specifically, we investigated the role of print-to-speech (feedforward) and speech-to-print (feedback) word consistency, derived across levels of granularity, in tasks of word naming, and visual and auditory lexical decision. We further contrasted these corpus-based measures of consistency with the implicit learning of these statistical regularities by neural network models to unveil which approach better accounts for human performance. Notably, the measures of consistency across various unit sizes were only moderately correlated with each other, while a composite of these measures accounted for variance in task performance over and above traditional word characteristics, like frequency and length. The main results can be summarized as follows: (1) robust feedforward and feedback consistency effects were obtained in word naming; (2) feedback consistency (but no feedforward consistency) effects were found in both visual and auditory lexical decision; (3) using a metric derived from neural network models (MSEs) as a proxy to consistency, both feedforward and feedback consistency effects were found across all three human tasks.

With regard to the first finding from Study 1 on word naming, the present results align with previous studies of quasi-regular orthographies such as English, where the rime’s consistency has been found to be a salient unit in reading monosyllabic words (De Cara & Goswami, 2002; Treiman & Kessler, 1995; Ziegler & Goswami, 2005), and onset consistency has been reported as a reliable predictor of word recognition (Balota et al., 2004; Treiman et al., 1995; Yap & Balota, 2009). While these sublexical units of onset-rime structure are important in early reading development, being accessible to children prior to their ability to reliably access phonemes (Goswami & Bryant, 1990; Treiman, 1992), the present results show that other sublexical units contribute more to adult word reading. Specifically, the onset-vowel-coda structure’s consistency was shown in the dominance analysis to account for more variance in adult naming times, and this was particularly the case for feedback consistency. Consistency measured at the rime and onset level had thus far led to disagreement regarding the effects of feedback consistency on word naming and visual recognition. For example, Balota et al., (2004) reported that feedback consistency of both the onset and rime affected naming latencies with results being more robust for naming than for lexical decision, while the opposite pattern was observed by Ziegler, Montant, and Jacobs (1997a). Later studies of visual lexical decision reported no feedback consistency effects (Kessler, Treiman, & Mullennix, 2008; Peereman, Content, & Bonin, 1998; Ziegler, Petrova, & Ferrand, 2008). One possible factor that may account for such conflicting results is how feedback consistency was defined or measured, as most of these previous studies have treated feedback consistency as a binary measure: If the rhyme spelling of a word is pronounced differently in other words, then the word is considered as inconsistent (e.g., Balota et al., 2004; Lacruz & Folk, 2004; Peereman et al., 1998; Stone et al., 1997; Ziegler et al., 1997a). Another concern is that the rime may not be the only unit that is relevant to pronunciation (Jared et al., 1990), as previous studies have shown that the pronunciation of vowels can vary systematically with the identity of the preceding consonant (Treiman, Kessler, & Bick, 2003; Treiman, Kessler, Zevin, Bick, & Davis, 2006). By taking all unit sizes into account here, the current studies show that feedback consistency reliably predicted word naming performance, as well as lexical decision seen in study 2, albeit with smaller effects. Considering the issues with defining consistency narrowly at one level of granularity, it is important for future studies to examine consistency at various grain sizes and treat it as a continuous variable with graded effects (e.g., Treiman et al., 1995; Jared et al., 1990).

With regard to the second main finding from Study 2, feedforward consistency did not impact lexical decision times as it did word reading times. This contradicts our initial prediction that visual lexical decision, but not auditory lexical decision, would depend in part on orthography-to-phonology consistency, with slower responses to printed words that could be pronounced in different ways, even though the task only requires lexical confirmation and not articulation of the word. While some investigators had previously argued that lexical decision should not show feedforward consistency effects since the task requires no pronunciation or reliance on phonology (Jared et al., 1990), this reasoning opposes the dynamic systems framework that suggests there are interactive connections between orthographic and phonological units (e.g., Stone & Van Orden, 1994; Van Orden & Goldinger, 1994). Further, studies have provided evidence of feedforward consistency effects in lexical decision tasks (e.g., Yap & Balota, 2009; Balota et al., 2004; Stone et al., 1997; Ziegler et al., 1997a). If information flows not only from spelling to sound but also from sound back to spelling, one would expect to find not only feedback but also feedforward consistency effects in lexical decision tasks. However, neither the composite measure of feedforward consistency (in the hierarchical regression models) nor any of the feedforward measures across different grain sizes (from the dominance analysis) showed significant contribution to lexical decision response times. On the other hand, our prediction that feedback consistency would contribute to auditory lexical decision exclusively was partially supported. Only the feedback composite and not the feedforward composite contributed to auditory lexical decision times, similar to the results for visual lexical decision. Thus, phonology-to-orthography feedback consistency comes into play when adult readers either hear or see a stimulus word/pseudoword. In contrast to these composite consistency measures derived from the corpus, a consistency metric derived from the PDP neural network model in Study 3 revealed both feedforward and feedback consistency effects in both lexical decision tasks, regardless of modality.

Regarding the third finding from Study 3, MSE-derived estimates of feedforward word consistency accounted for more variance than the corpus-derived measures of feedforward consistency in all three tasks. In particular, the effect of feedforward composite consistency that was observed in the naming task disappeared when MSEs were added to the final regression model, suggesting that the print-sound information that visual word naming relied upon was not fully captured in the composite consistency measure. In comparing feedback consistency effects, the corpus-derived composite main effect on auditory lexical decision times disappeared when MSEs were added to the final model. Whereas this was not observed in the two visual modality tasks, naming and lexical decision, where the corpus-based feedback consistency effects remained significant, though weaker, when MSEs were added. This suggests that the data-driven MSEs can fully account for the conventional consistency measures computed based on parts of words, at least for auditory lexical decision. Together, the present study demonstrates that the data-driven MSE obtained from a bidirectional PDP model could be a more reliable estimator of print-sound and sound-print consistency than that based on the properties of a word’s neighborhood. Moreover, token-based MSE estimates best predict performance of adult readers. This is likely due to accounting for the frequency that readers come across a given word, thus indicating that consistency should take token frequency into account.

To summarize, the present work demonstrated how consistency can be computed over different parts of the word as a continuous composite measure, and can show stable feedback consistency effects across naming, visual and auditory lexical decision tasks. The robust feedforward and feedback consistency effects observed across the three tasks in Study 3 indicate interactivity between a word’s phonology and orthography in word-recognition tasks, consistent with the hypotheses made in some previous studies (e.g., Coltheart et al., 2001; Van Orden & Goldinger, 1994; Van Orden et al., 1990). These findings support several predictions made based on interactive networks (e.g., Interactive Activation model, (McClelland & Rumelhart, 1981); Parallel-Distributed Processing model, Seidenberg and McClelland (1989)). First, feedback consistency effects can be found across naming and lexical decision tasks (e.g., Lacruz & Folk, 2004; Pecher, 2001), indicating that phonology is involved in the process of word recognition. Second, consistency matters in both orthography-to-phonology and phonology-to-orthography directions, supporting the cross-code consistency account proposed by Grainger et al., (2005). Third, feedback consistency effects can occur in both visual (e.g., Stone et al., 1997; Ziegler et al., 1997a) and spoken word recognition (e.g., Ziegler and Ferrand, 1998; Ventura et al., 2004; Miller & Swick, 2003).

### Implications for models of reading

The present results are novel with regard to quantifying quasi-regularity in the orthography and phonology mapping for English words through the implicit learning process of a neural network. Moreover, mechanisms involved in word recognition may be better elucidated by the current neural network models, which include fully bi-directional links amongst units in the three layers: orthographic, hidden, and phonemic. This contrasts with previous recurrent networks (e.g., Plaut et al., 1996) which simulated the reading direction flow of information (orthography to phonology) where feedback connections were restricted to phonology-to-hidden units. Our models encapsulate a functional ’reader’ who is not only versed in reading but also spelling and writing—thus bidirectional information flows in the reading direction, but also the spelling direction for our models. We assume a close relation between reading and spelling processes which mutually affect each other, such that naming a word using orthography-to-phonology links also involves feedback of the retrieved phonological representation to verify the word’s orthographic form, or spelling.

Specifically, we utilized one of the two main classes of computational reading models—the PDP model (Harm & Seidenberg, 2004), which makes no distinction between lexical and sublexical processing, instead instantiating phonology-orthography mappings through largely emergent co-activated patterns across granularities. We used back-propagation through time (BPTT; ; Werbos, 1990) algorithm for training our recurrent network where the states of units in the network change smoothly over time in response to influences from other units. When the activity in the input layer at time *t* − 1 is feedforwarded, all hidden units receive the corresponding input at time *t* through the feedforward orthography-hidden connections. In a similar fashion, when hidden layer activity at time *t* − 1 is feedforwarded through connections between the hidden and output layers, all output units at time *t* are affected. Even when a model is trained only on a reading task, the existence of feedback connections would cause the activity in the output layer at time *t* − 1 to influence the hidden activity at time *t*. Once all timesteps have passed, a single backward pass through all of the ticks is performed and error derivatives are injected to update the connection weights.

Without advocating for one or the other main classes of computational reading models (PDP or DRC, dual-route cascaded model, Coltheart, Curtis, Atkins, and Haller, 1993; Coltheart et al., 2001)—we draw distinctions between these architectures based on the granularity at which orthographic and phonological representations are mapped. In contrast to PDP, DRC models do distinguish lexical from sublexical processing, by instantiating a set of pre-determined print-to-speech correspondence rules at the sublexical level and interconnected lexicons (for orthographic and phonological representations) at the lexical level. This accounts for reading of both irregular and regularly spelled words. To account for the feedforward consistency effect on naming, an architecture combining the DRC with a PDP network for sublexical processing was developed as the Connectionist Dual Process model (CDP++, Perry et al., 2007; 2010). The sublexical network involves two layers that are trained to associate graphemes with phonemes through exposure to real words, just as in PDP, however the mapping process is feedforward, and there are conflicting results as to whether the mechanism accounts for feedback effects (Ziegler et al., 2008; Yap & Balota, 2009). This challenges the idea of a bidirectional coupling as necessarily involving a feedback mechanism in the sublexical route for feedback consistency effects to manifest.

Our PDP model was inspired by the resonance theory of word perception put forward by Van Orden and Goldinger (1994), whereby orthographic representations communicate bidirectionally with both phonological and semantic representations as the initial activation spreads across the network following presentation of a printed word stimulus. In such an interactive model, both feedforward and feedback consistency of an input determine how fast and stable activation propagates through the network (see also the cross-code consistency account proposed by Grainger et al., (2005)).

When our PDP model explicitly implemented bidirectional connections between orthography and phonology, the network’s error during reading aloud (i.e., orthography-to-phonology) was higher for more feedback-inconsistent words. This feat suggests a resonance effect such that word naming reorganizes both feedforward and feedback connections in a way that optimizes the subregularities between the orthographic and phonological layers in both ways. Such optimization still has to consider the quasi-regular nature of mappings. When a feedback inconsistent word is presented to the bidirectional reading model in a naming task during the training phase, the activated phonological representations will, via feedback connections, re-activate orthographic representations for several word bodies. These orthographic representations will constrain each other and the competition will slow the learning process for feedback inconsistent words, resulting in a higher reading MSE. In the context of a lexical decision task where semantic knowledge is necessary, the activated phonological and semantic representations in a triangle model (e.g., Plaut et al., 1996) will similarly re-activate the orthographic representations via feedback connections. Although our current model lacked a semantic layer to capture such interactions between orthography-semantic and phonology-semantic levels, the present results indicate that the resonance between orthographic and phonological units plays a role in word recognition and, across tasks, this bidirectional activation between orthography and phonology is likely captured in several different grain sizes of representation that are difficult to measure as one composite variable.

### Implications for theories of reading and reading development

Our general finding of word consistency effects on adult word recognition suggests that these emerge over different levels of granularity, and they are bidirectional, from print to sound and vice versa. This has several implications for developing readers. First, because consistency effects are present in skilled adult readers, it is important to identify the degree of consistency for words that are part of early literacy instruction. As young children have to acquire print-sound correspondences, in many cases on an implicit learning basis, their exposure to printed words must facilitate this learning process. In the interest of ranking words by their degree of consistency, the extant literature has focused on different definitions of consistency—from rime patterns, to single graphemes. Thus, an accounting of consistency across granularities would be a more useful resource.

Secondly, in spite of a general consensus that reading and writing skills tend to co-develop in young children, only a few theories of reading development directly address this dynamic, interactive process (Frith, 1985; Bosman and Van Orden, 1997; Lerkkanen, Rasku-puttonen, Aunola, & Nurmi, 2004; Kim, Petscher, Wanzek, & Al Otaiba, 2018). There are more recent calls for an integrated science of reading and writing (Graham, 2020). A better understanding of the joint development of these literacy skills may contribute directly to how teachers can plan lessons in spelling such that both letter(s)-sound and sound-letter(s) patterns can be reinforced, along with higher level literacy skills (Graham, 2020). Educators could avail themselves of the consistency measures obtained in the current studies for the purpose of identifying specific sets of words that are more challenging to learn to read and spell, or to rank words according to their consistency metrics and use this as a basis for when to introduce words into the literacy curriculum. Reading experts have long recognized that teaching spelling to early readers helps them develop more robust mental representations (Moats, 2005; Snow & Juel, 2005; Andrews, Veldre, & Clarke, 2020).

While the current study examined effects of feedback and feedforward consistency and bidirectional orthographic-phonological resonance on adult reading performance, future research could take a similar approach to examine developmental trajectories according to these effects. Using a neural network modelling approach would be fruitful in understanding related phenomena of consistency, word frequency and age of acquisition effects in a development model where lexical representations and neighborhood effects would be dynamic. Capturing these effects in a development model could flesh out print exposure mechanisms such as lexical tuning (Castles, Davis, Cavalot, & Forster, 2007), or lexical restructuring with increased vocabulary (Goswami, 2000; Walley, Metsala, & Garlock, 2003). This work would have interdisciplinary relevance to various fields at the intersection of cognitive science and education.

## Supplementary information

### Uni- vs. bi-directional models

**Fig. 10:**
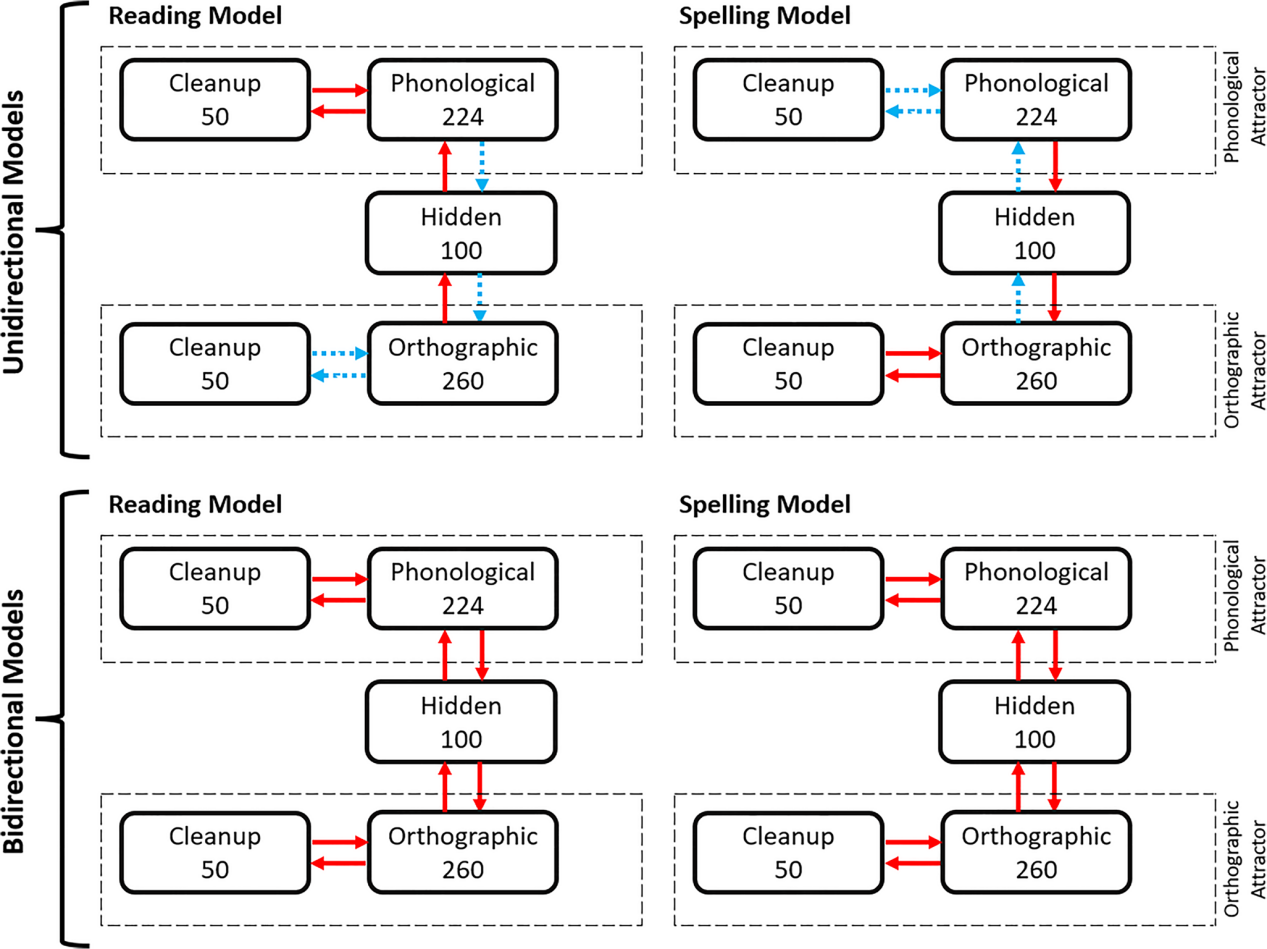
Architecture of the reading and spelling connectionist models implemented. The *top* and *bottom panels* depict unidirectional and bidirectional models, respectively. *Red solid lines* indicate trainable weights; and *blue dashed lines* indicate frozen weights

We tested two sets of models to determine if bidirectional connections are needed in order to capture orthography-to-phonology (O2P) or phonology-to-orthography (P2O) mapping consistency when trained on a unidirectional task (i.e., reading or spelling). All models had the same architecture so that their results are comparable, and differed only in whether the task-irrelevant weights were frozen at its initial value during training (Fig. 10). In the first sets of unidirectional models, both the reading and spelling models had their task-irrelevant P2O and O2P’s weights frozen, respectively, to simulate the dynamic of a unidirectional network. In the second sets of bidirectional models, however, none of the weights were frozen and models were allowed to change the weights in both O2P and P2O directions during training.

Across all three data sets, results showed that MSEs extracted from bi-directional models yield a lower AIC and thus a better fit for human RTs than that from uni-directional models, when compared in the same direction (i.e., feedback vs. feedback and feedforward vs. feedforward MSEs) (Tables 15, 16, and 17). This shows that bi-directional connections are necessary for PDP models to maximally extract latent quasi-regularities in spelling–sound and sound–spelling mappings.

**Table 15:** Comparison of using uni- and bi-directional models’ MSEs to predict visual naming performance

Model	beta	df	AICc	Delta AICc
FF_Bi_MSE	0.163	10	10116.76	0.00
FB_Bi_MSE	0.177	10	10130.47	13.72
FB_Uni_MSE	0.175	10	10134.66	17.90
FF_Uni_MSE	0.140	10	10156.20	39.45
Baseline		9	10270.75	154.00

**Table 16:** Comparison of using uni- and bi-directional models’ MSEs to predict visual lexical decision performance

Model	beta	df	AICc	Delta AICc
FB_Bi_MSE	0.116	10	9928.41	0.00
FB_Uni_MSE	0.113	10	9932.16	3.75
FF_Bi_MSE	0.076	10	9957.40	28.99
FF_Uni_MSE	0.068	10	9964.36	35.95
Baseline		9	9991.33	62.92

**Table 17:** Comparison of using uni- and bi-directional models’ MSEs to predict auditory lexical decision performance

Model	beta	df	AICc	Delta AICc
FB_Uni_MSE	0.056	10	11876.09	0.00
FB_Bi_MSE	0.040	10	11881.22	5.13
FF_Bi_MSE	0.028	10	11883.15	7.06
FF_Uni_MSE	0.026	10	11883.74	7.65
Baseline		9	11884.73	8.64

### Individual hierarchical regression analyses for visual naming task

In Table 10, the regression analyses indicate that both FF_MSE and FB_MSE contribute similarly in terms of magnitude to human naming performance. However, in the previous regression analyses of composite measures (refer to Table 4), FB_Composite accounted for a greater proportion of the variance compared to the FF_Composite score. To investigate the reason for this disparity between MSE and composite measures, we conducted four additional hierarchical regression analyses for the visual naming task. These analyses followed an identical regression model in step 1 and included one of the four measures of interest (i.e., FF_MSE, FB_MSE, FF_Composite, FB_Composite) in step 2.

Interestingly, when FB_Composite was included in the model, the previously significant OrthND effect became nonsignificant in step 2 (Table 18). This change in the OrthND effect, however, was not observed when FF_Composite, FB_MSE, or FF_MSE were added to the same step-1 model (Tables 19, 20, 21). These findings suggest that FB_Composite is associated with OrthND and reflects the combined influence of OrthND and its own effect in the step-2 model. When FB_Composite was excluded in step 1, OrthND captured the partial effect of FB_Composite and therefore remained significant.

**Table 18:** Complete hierarchical regression of FB_Composite predicting visual naming task performance

Predictor	*beta*	*beta* 95% CI	Fit
Step 1			
Frequency	− 0.30**	[− 0.33, − 0.28]	
Voice	− 0.26**	[− 0.29, − 0.23]	
Onset_Coding	0.08**	[0.05, 0.10]	
Word_Length	0.28**	[0.24, 0.33]	
Num_Phones	− 0.16**	[− 0.20, − 0.12]	
OrthND	− 0.11**	[− 0.15, − 0.06]	
PhonND	0.04	[− 0.01, 0.08]	
			*R*^2^ =.330** 95% CI[.31, .35]
Step 2			
Frequency	− 0.25**	[− 0.27, − 0.22]	
Voice	− 0.26**	[− 0.29, − 0.24]	
Onset_Coding	0.08**	[0.05, 0.11]	
Word_Length	0.22**	[0.17, 0.26]	
Num_Phones	− 0.07**	[− 0.12, − 0.03]	
OrthND	− 0.01	[− 0.05, 0.03]	
PhonND	− 0.05*	[− 0.09, − 0.01]	
FB_Composite	− 0.24**	[− 0.27, − 0.21]	
			*R*^2^ =.371** 95% CI[.35, .39] *Δ* *R*^2^ =.041** 95% CI[.03, .05]

**Table 19:** Complete hierarchical regression of FF_Composite predicting visual naming task performance

Predictor	*beta*	*beta* 95% CI	Fit
Step 1			
Frequency	− 0.30**	[− 0.33, − 0.28]	
Voice	− 0.26**	[− 0.29, − 0.23]	
Onset_Coding	0.08**	[0.05, 0.10]	
Word_Length	0.28**	[0.24, 0.33]	
Num_Phones	− 0.16**	[− 0.20, − 0.12]	
OrthND	− 0.11**	[− 0.15, − 0.06]	
PhonND	0.04	[− 0.01, 0.08]	
			*R*^2^ =.330** 95% CI[.31, .35]
Step 2			
Frequency	− 0.28**	[− 0.31, − 0.26]	
Voice	− 0.26**	[− 0.28, − 0.23]	
Onset_Coding	0.08**	[0.06, 0.11]	
Word_Length	0.27**	[0.23, 0.32]	
Num_Phones	− 0.13**	[− 0.18, − 0.09]	
OrthND	− 0.10**	[− 0.15, − 0.06]	
PhonND	0.04	[− 0.00, 0.08]	
FF_Composite	− 0.12**	[− 0.15, − 0.10]	
			*R*^2^ =.345** 95% CI[.32, .36] *Δ* *R*^2^ =.015** 95% CI[.01, .02]

**Table 20:** Complete hierarchical regression of FB_MSE predicting visual naming task performance

Predictor	*beta*	*beta* 95% CI	Fit
Step 1			
Frequency	− 0.30**	[− 0.33, − 0.28]	
Voice	− 0.26**	[− 0.29, − 0.23]	
Onset_Coding	0.08**	[0.05, 0.10]	
Word_Length	0.28**	[0.24, 0.33]	
Num_Phones	− 0.16**	[− 0.20, − 0.12]	
OrthND	− 0.11**	[− 0.15, − 0.06]	
PhonND	0.04	[− 0.01, 0.08]	
			*R*^2^ =.330** 95% CI[.31, .35]
Step 2			
Frequency	− 0.23**	[− 0.26, − 0.20]	
Voice	− 0.26**	[− 0.29, − 0.23]	
Onset_Coding	0.09**	[0.06, 0.11]	
Word_Length	0.23**	[0.19, 0.28]	
Num_Phones	− 0.12**	[− 0.16, − 0.07]	
OrthND	− 0.07**	[− 0.11, − 0.03]	
PhonND	− 0.03	[− 0.07, 0.01]	
FB_MSE	0.18**	[0.15, 0.21]	
			*R*^2^ =.352** 95% CI[.33, .37] *Δ* *R*^2^ =.022** 95% CI[.02, .03]

**Table 21:** Complete hierarchical regression of FF_MSE predicting visual naming task performance

Predictor	*beta*	*beta* 95% CI	Fit
Step 1			
Frequency	− 0.30**	[− 0.33, − 0.28]	
Voice	− 0.26**	[− 0.29, − 0.23]	
Onset_Coding	0.08**	[0.05, 0.10]	
Word_Length	0.28**	[0.24, 0.33]	
Num_Phones	− 0.16**	[− 0.20, − 0.12]	
OrthND	− 0.11**	[− 0.15, − 0.06]	
PhonND	0.04	[− 0.01, 0.08]	
			*R*^2^ =.330** 95% CI[.31, .35]
Step 2			
Frequency	− 0.26**	[− 0.29, − 0.24]	
Voice	− 0.26**	[− 0.28, − 0.23]	
Onset_Coding	0.09**	[0.06, 0.11]	
Word_Length	0.29**	[0.25, 0.34]	
Num_Phones	− 0.16**	[− 0.21, − 0.12]	
OrthND	− 0.08**	[− 0.13, − 0.04]	
PhonND	0.03	[− 0.01, 0.07]	
FF_MSE	0.16**	[0.14, 0.19]	
			*R*^2^ =.354** 95% CI[.33, .37] *Δ* *R*^2^ =.024** 95% CI[.02, .03]
